# A Culture-Independent Approach to Unravel Uncultured Bacteria and Functional Genes in a Complex Microbial Community

**DOI:** 10.1371/journal.pone.0047530

**Published:** 2012-10-17

**Authors:** Yun Wang, Yin Chen, Qian Zhou, Shi Huang, Kang Ning, Jian Xu, Robert M. Kalin, Stephen Rolfe, Wei E. Huang

**Affiliations:** 1 Kroto Research Institute, University of Sheffield, Sheffield, England, United Kingdom; 2 School of Life Sciences, University of Warwick, Coventry, England, United Kingdom; 3 BioEnergy Genome Centre, Chinese Academy of Sciences Key Laboratory of Biofuels and Shandong Key Laboratory of Energy Genetics, Qingdao Institute of BioEnergy and Bioprocess Technology, Chinese Academy of Sciences, Qingdao, China; 4 David Livingstone Centre for Sustainability, Strathclyde University, Glasgow, Scotland, United Kingdom; 5 Department of Animal and Plant Sciences, Alfred Denny Building, University of Sheffield, Sheffield, England, United Kingdom; University of Georgia, United States of America

## Abstract

Most microorganisms in nature are uncultured with unknown functionality. Sequence-based metagenomics alone answers ‘*who*/*what are there?’* but not ‘*what are they doing and who is doing it and how?’.* Function-based metagenomics reveals gene function but is usually limited by the specificity and sensitivity of screening strategies, especially the identification of clones whose functional gene expression has no distinguishable activity or phenotypes. A ‘biosensor-based genetic transducer’ (BGT) technique, which employs a whole-cell biosensor to quantitatively detect expression of inserted genes encoding designated functions, is able to screen for functionality of unknown genes from uncultured microorganisms. In this study, BGT was integrated with Stable isotope probing (SIP)-enabled Metagenomics to form a culture-independent SMB toolbox. The utility of this approach was demonstrated in the discovery of a novel functional gene cluster in naphthalene contaminated groundwater. Specifically, metagenomic sequencing of the ^13^C-DNA fraction obtained by SIP indicated that an uncultured *Acidovorax* sp. was the dominant key naphthalene degrader *in-situ*, although three culturable *Pseudomonas* sp. degraders were also present in the same groundwater. BGT verified the functionality of a new *nag2* operon which co-existed with two other *nag* and two *nah* operons for naphthalene biodegradation in the same microbial community. Pyrosequencing analysis showed that the *nag*2 operon was the key functional operon in naphthalene degradation *in-situ,* and shared homology with both *nag* operons in *Ralstonia* sp. U2 and *Polaromonas naphthalenivorans* CJ2. The SMB toolbox will be useful in providing deep insights into uncultured microorganisms and unravelling their ecological roles in natural environments.

## Introduction

Bacteria account for approximately half of the total carbon of the global biomass [1] and play fundamental roles in biogeochemical cycles (e.g. C and N) [2]. Bacteria provide a free service worth trillions of dollars to maintain and restore ecosystems - cleaning water and soil, and maintaining soil fertility [3]. However, the vast majority of bacteria (>99%) present in natural environments have not yet been cultured [4], [5], [6], [7], [8]. These uncultured bacteria are often referred to as a ‘black box’ containing a ‘hidden’ community that represents an untapped genetic resource encoding novel and valuable catalysts, enzymes and building blocks for industry and medicine [9], [10], [11], [12]. In addition, whilst cultivation of pure isolates enables the detailed study of bacterial physiology, it is often more desirable to study microbial genetic function and ecological roles *in-situ*. Therefore, the development of culture-independent approaches will provide a global view of the bacterial community, help predict ecosystem functioning and lead to further understanding of bacterial evolution [13], [14].

Metagenomics circumvents the cultivation issue by extracting total DNA from an environmental sample, followed either by direct sequencing (sequence-based metagenomics) or cloning into a culturable model organism for functional analysis (function-based metagenomics) [15], [16], [17]. Advances in next generation DNA sequencing technologies (e.g. 454 pyrosequencing) [18], [19], [20] have provided large data sets for sequence-based metagenomics addressing questions such as ‘*who*/*what are there?’*. However, it is also important to identify gene functions and answer ‘*what are they doing* and *who is doing it and how?’*. Since metagenomics treats a microbial community as a single data-set, a metagenomic approach alone makes limited links between specific microorganisms and their ecological functions in a biological context [21], [22], [23]. One approach is to use bioinformatic-derived predictions to annotate gene functions but this fails to predict function for more than 30% of sequenced genes [24]. Function-based metagenomics is often hampered by the limits of selective screening strategies, especially for the identification of functional genes whose expression has no detectable activity or distinguishable phenotypes. Therefore, identification of gene function is one of the key challenges in the post-genomic era.

To address these challenges, we have developed a culture-independent SMB toolbox that integrates Stable isotope probing (SIP)-enabled Metagenomics with a Biosensor-based gene transducer (BGT) technique. SIP provides the link between bacteria and the metabolism of stable isotope (e.g. ^13^C, ^15^N) labeled substrates. The ^13^C-DNA fraction obtained by SIP provides a significantly-reduced but relevant gene-pool for metagenomic analysis [25]. Sequencing of the ^13^C-DNA fraction specifically reveals microorganisms whose activities are involved in metabolism of the ^13^C-labelled substrate. BGT identifies and verifies functional genes involved in the metabolism of a specific target compound.

**Figure 1:**
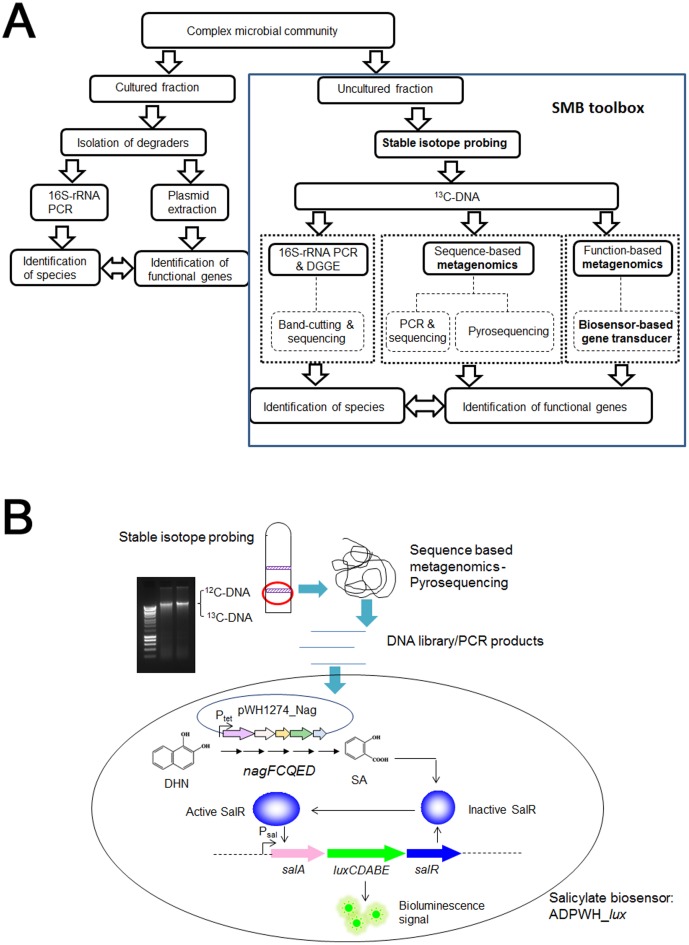
A. Schematic of a toolbox to dissect microbial community structure and their functional genes in a complex community. The culture-independent SMB toolbox comprises stable isotope probing, metagenomic sequencing and biosensor-based gene transducer. **B.** **Schematic of the biosensor-based gene transducer.** This approach can be used to screen for gene encoded functions for the metabolism of molecules with no distinguishable activity or phenotypes.

In this study, we chose a well-characterised naphthalene contaminated groundwater site [26], [27] to demonstrate the use of the SMB toolbox. We employed both culture-dependent and culture-independent methods, as illustrated in Fig. 1A, to provide a holistic picture of the microbial community in the groundwater. For the cultured fraction, traditional methods including cultivation, conjugation and plasmid extraction were used to isolate and identify putative naphthalene degraders and their functional genes. For the uncultured fraction, the SMB toolbox was applied to identify uncultured but active naphthalene degraders and to investigate their functional genes *in-situ* (Fig. 1A and 1B).

## Results

Since the active members for naphthalene degradation *in-situ* are unknown, we employed both culture dependent and independent approaches to investigate the microbial community by splitting a naphthalene-contaminated groundwater sample into cultured and uncultured fractions. For cultured fraction, we isolated three *Pseudomonas* spp. that can grow by using naphthalene as a sole carbon source and identified two types of *nah* operons located on plasmids in the hosts. For uncultured fraction, we applied SMB to study the active bacteria in the microbial community. We found that an uncultured *Acidovorax* sp. was responsible for naphthalene degradation *in-situ.* Metagenomic sequencing and the BGT technique revealed that one mosaic pattern *nag* and two other *nag* operons were the active functional operons for naphthalene biodegradation in the groundwater.

### Cultured Fraction: Naphthalene Degradation Genes were Located on Conjugative Plasmids of Isolated Naphthalene Degraders

Conventional cultivation approaches were used for preliminary investigations of naphthalene-degrading microorganisms. *Pseudomonas fluorescens* (WH2) and two *Pseudomonas putida* (WH1 and WH3) naphthalene degraders were isolated from the contaminated groundwater. All three isolates could grow on the minimal medium (MM) [28] with naphthalene as a sole carbon source. Conjugation between donor bacteria: *P. putida* WH1, WH3, *P. fluorescens* WH2 and *P. putida* NCIB9816 (used as a positive control) [29] and recipient *P. putida* UWC1 [30] separately indicated that the naphthalene degradation genes were located on conjugative plasmids in *P. putida* WH1, WH3, *P. fluorescens* WH2. The conjugation frequencies for *Pseudomonas* WH1, WH2, WH3 and NCIB9816 were respectively 2.03±0.12×10^−7^, 2.33±0.73 ×10^−7^, 1.93±0.14 ×10^−7^ and 4.33±1.2 ×10^−8^ transformants/recipient.

Restriction enzyme digestion patterns indicated that the *P. putida* WH1 and WH3 plasmids had the same structure whilst *P. fluorescens* WH2 differed (Fig. S1). DNA sequence analysis showed that the genes encoding the salicylate hydroxylase (NahG) and the transcriptional regulator (NahR) on the plasmids of *P. putida* WH1 and WH3 were identical to those on pDTG1 in *P. putida* NCIB 9816 [31] despite the different plasmid structures (Fig. S1). The *nahG* and *nahR* genes in *P. fluorescens* WH2 were identical to those in the chromosome of *Pseudomonas stutzeri* AN10 [32].

### Uncultured Fraction: SIP Enriched ^13^C-DNA Revealed *Acidovorax* sp. as a Key Naphthalene Degrader

The use of SIP requires an appropriate incubation period that sufficient for nucleic acids of active degraders to become isotopically enriched. However, the physical (e.g. temperature) and chemical (e.g. naphthalene concentration) conditions of this incubation may alter the microbial community structure. Therefore the impact of altering incubation conditions on the microbial community was investigated. Denaturing gradient gel electrophoresis (DGGE) of 16S rRNA genes was used to assess microbial community structure and diversity in the groundwater. The DGGE profiles of microcosms incubated in darkness at 14°C with 3.8 µM naphthalene (ambient concentration) for 168 h were similar to the profile from the native groundwater (Fig. S2). However, changing either the temperature of incubation (from 14 to 20°C) or naphthalene concentration (from 3.8 to 30 µM) significantly altered both bacterial diversity and relative abundance in the microbial community (Fig. S2). This demonstrates the importance of incubation replicating, as far as possible, field conditions (dark, 14°C and 3.8 µM naphthalene) in order to identify the *in-situ* degraders.

The incubation period must also be as short as possible to minimize ^13^C-cross-labeling of other community members with ^13^C-metabolites released from the primary degraders. Aerobic degradation of ^13^C-naphthalene produced salicylate in the microcosms which was monitored using a salicylate biosensor ADPWH_*lux* as a rapid detection tool [28]. A peak of salicylate production was found to appear after 120 h in the microcosms which returned to the background level at 144 h (Fig. S3), suggesting that active aerobic naphthalene biodegradation had occurred during this period. Total nucleic acid containing ^12^C- and ^13^C-DNA was extracted from the microcosms after 120 h and purified. After equilibrium density gradient ultracentrifugation, the ‘heavy’ ^13^C-DNA fractions were recovered, which contained genomic DNA of the active naphthalene degraders *in-situ*.

DGGE analysis of the ^13^C-DNA fraction showed that band B (labeled as B1, B2, B3 respectively in the three ^13^C-DNA fractions in Fig. S4) was dominant in the ^13^C-DNA fraction (Fig. S4). Sequence analysis of band B suggested that it was derived from *Acidovorax* sp. (designated as *Acidovorax* sp. WH), consistent with our previous reports [26]. The result suggested that an uncultured *Acidovorax* sp., rather than the cultured *Pseudomonas* sp., played a key role in naphthalene biodegradation *in-situ* even though they co-existed in the same groundwater. It is not unusual that uncultured *Acidovorax* sp. was found to play an important role in aromatic hydrocarbon biodegradation, as it has also been found in other studies [33].

**Figure 2:**
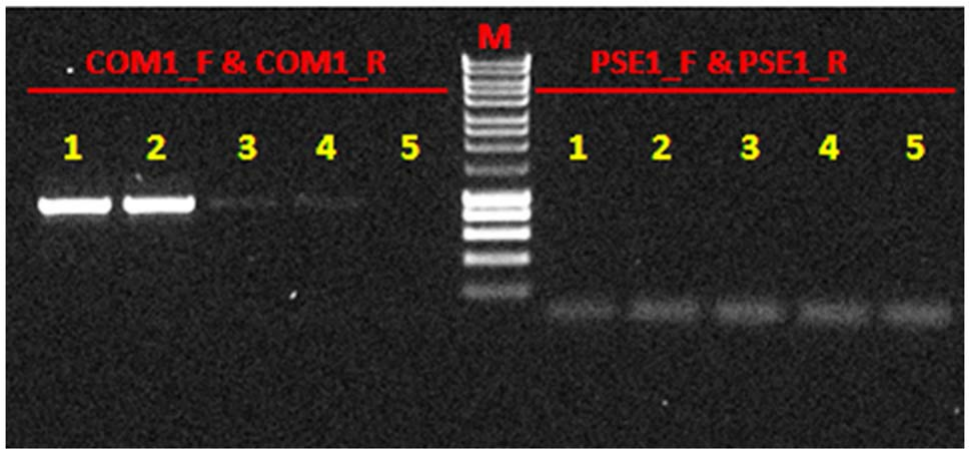
Naphthalene dioxygenase (NDO) PCR products. Two pairs of degenerate primers, COM1_F&COM1_R (*Comamonas*-type), and PSE1_F&PSE1_R (*Pseudomonas*-type) (Table 1) and different DNA templates: 1, 2 - ^13^C-DNA; 3, 4 - ^12^C-DNA; 5– blank control were used. M - DNA molecular weight Ladder.

Strong PCR products were obtained with *Comamonas*-type naphthalene dioxygenase (NDO) primers in ^13^C-DNA fractions but no PCR products were observed using *Pseudomonas*-type NDO primers (Fig. 2). This result is in a good agreement with the 16S-rRNA analysis above which indicates that under the field-site conditions the functional genes for *in-situ* naphthalene biodegradation were from *Comanonadaceae* spp. rather than the isolated *Pseudomonas* spp. The PCR products were cloned into a plasmid vector. Ten were chosen at random and sequenced. Of these sequences, 20% were identical to *Comamonas*-type NDO in the *nag* operon hosted by *Ralstonia* sp. U2, and 80% were hybrids of *Ralstonia* sp. U2 and *P. naphthalenivorans* CJ2.

**Table 1:** Primers used in this study.

Primers	Sequence (5′→3′)	Reference
NagF1_For	TTCCCAGGAGACAACCCATG	This study
Nag_F	AGTTCATCACTGGCACCGTA	This study
NagD_Rev	TGAGGCGACAATGAACATGC	This study
NagSeq1_F	ATCGTTCTGGACGACGCTGAC	This study
NagSeq2_F	CAAGCCAGCAACTGTCATTG	This study
NagSeq3_F1	CGAAGATTTGGGTTACACACACC	This study
NagSeq3_F2	ACCTACAACCTGCCACAGATG	This study
NagSeq4_F	ATTCAAGGCGCATGGGTCATCA	This study
NagSeq5_F	GGATGCTGGACTTGGATCTGA	This study
GC338F	CGCCCGCCGCGCCCCCGCCCCGGCCCGCCGCCCCCGCCCACTCCTACGGGAGGCAGC	[60]
530R	GTATTACCGCGGCTGCTG	[60]
COM1_F	AAAAGAGTTGTACGGCGATG	[61]
COM1_R	ACGGTAGAATCCGCGATAGC	[61]
PSE1_F	AAAAGAGCTGTATGGCGAGT	[61]
PSE1_R	CCGATAGAAGCCACGATAACT	[61]
pWH1274_P12	CATGATCGCGTAGTCGATAG	This study
pWH1A1_1_P4R	AGTGCCACCTGACGTCTAAG	This study

### Pyrosequencing Analysis of Microorganisms and Functional Genes Involved in Naphthalene Degradation

**Figure 3:**
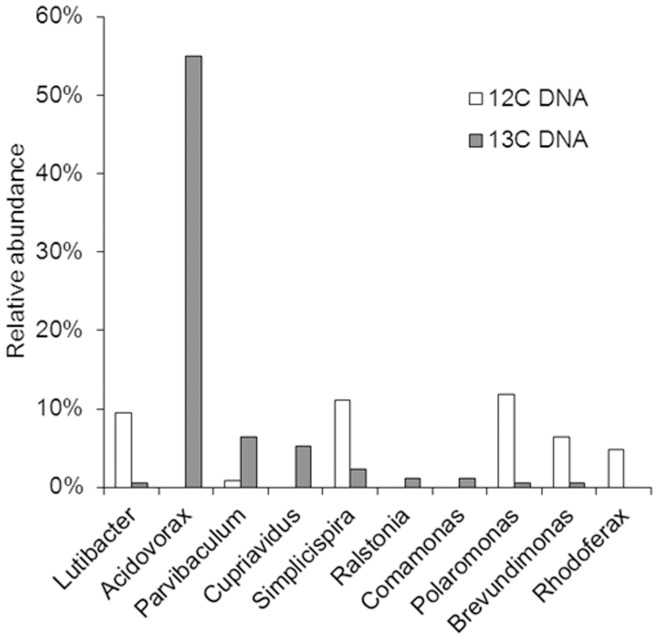
Taxonomic assignments of the microbial communities in the ^12^C- and ^13^C-DNA fractions revealed by pyrosequencing. Relative abundance of the species as a % of total 16S-rRNA reads is shown.

Pyrosequencing of the ^12^C- and ^13^C-DNA fractions produced separately 269,001 and 797,526 reads, ranging from 30 to 530 bp in length. Analysis of 16S- rRNA genes within the ^13^C-DNA fraction pyrosequencing data showed that *Acidovorax* sp. (55%) were dominant and *Ralstonia* sp. (1%) and *Polaromonas* sp. (0.1%) were also present (Fig. 3 and Fig. S5). None of these bacteria have yet been isolated using traditional culture-dependent methods. The 16S- rRNA gene of *Pseudomonas* sp. was not found in the ^13^C-DNA fraction. The 16S- rRNA gene sequences in the ^13^C-DNA fraction also included those with 100% identity to that of *Ralstonia* sp. U2 (1180–1450 bp in AF301897) and 99% identity to that of *Polaromonas naphthalenivorans* CJ2 (1014–1514 bp in NC_008781). These pyrosequencing reads include hypervariable regions of the 16S rRNA gene [34], suggesting that the naphthalene degraders revealed by SIP are similar to *Ralstonia* sp. U2 and *P. naphthalenivorans* CJ2 which have been reported previously to be naphthalene degraders.

Pyrosequencing assembly (DNA assembly file) recovered three assemblies: one 21,173 bp and one 25,765 bp assemblies each have >99.9% identity to the *nag* operon in *Ralstonia* sp. U2 (*nag*-U2) [35] and *Polaromonas naphthalenivorans* CJ2 (*nag*-CJ2) [36]; the third and dominant one designated as *nag2*, was a hybrid of *nag*-U2 and *nag*-CJ2. The *nag*-CJ2, *nag*-U2 and *nag2* separately account for 18.0% and 17.7% and 64.3% of the *nag*-related reads in the ^13^C-DNA fraction (Fig. S6 and Table S1). No *nah* operon was found in the ^13^C-DNA fraction. The *nag2* forms a mosaic pattern operon sharing homology with both *nag*-CJ2 and *nag*-U2 (Fig. S6 and Table S1).

### Biosensor-based Gene Transducer (BGT) Technique Confirmed a New Operon Carrying a Naphthalene Degradation Function

**Figure 4:**
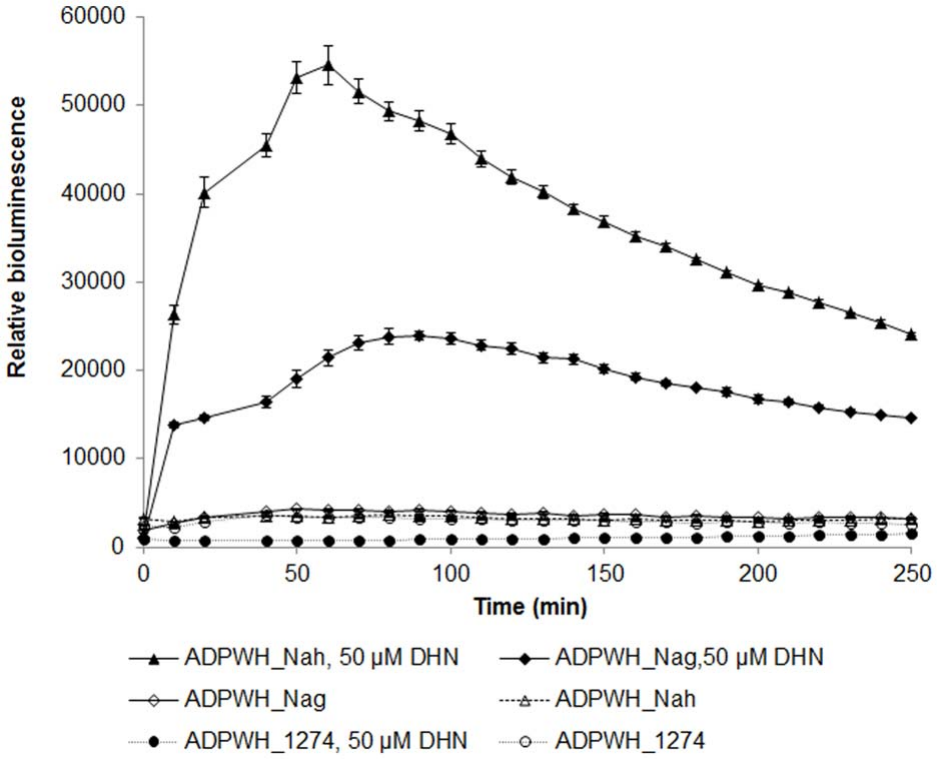
Bioluminescence of biosensor transducers incorporating DHN degradation genes. ADPWH_Nag containing a *nag*2 gene cluster was rapidly activated to show bioluminescence after the addition of 50 µM DHN, confirming the function of the gene cluster. No bioluminescence was detected in the absence DHN. The negative control ADPWH_1274 did not respond to DHN. ADPWH_Nah containing a *nah* gene cluster was used as a positive control.

The *nagFCQED* gene cluster was amplified from the ^13^C-DNA fraction using primers designed on the basis of the *nag* operon sequence (Table 1). This was then ligated into the plasmid pWH1274 and transformed into the salicylate biosensor strain ADPWH_*lux* (Huang et al., 2005) to produce a strain designated as ADPWH_Nag (Table 2). The well-characterised gene cluster *nahFCQED* was amplified by PCR from the naphthalene degradation plasmid pDTG1 in *P. putida* NCIB9816 [31] and inserted into pWH1274 in the same manner to produce ADPWH_Nah for the use as a positive control (Table 2). The plasmid pWH1274 is an *E. coli - Acinetobacter* shuttle [37] vector and the *nagFCQED* or *nahFCQED* was inserted into the BamHI site of pWH1274 under the control of a constitutive promoter P_tet_. ADPWH_*lux* containing the plasmid pWH1274 without inserts (ADPWH_1274) was used as a negative control (Table 2). The enzymes encoded by the targeted gene cluster should convert the substrate 1,2-dihydroxynaphthalene (DHN) into salicylate, which activates the P_sal_ promoter and triggers the expression of bioluminescence in ADPWH_*lux* (Fig. 1B). As shown in Fig. 4, both ADPWH_Nag and ADPWH_Nah were induced to express bioluminescence within 5 min in the presence of 50 µM DHN, indicating that the *nagFCQED* gene cluster was functional and its expression in ADPWH_*lux* was able to convert DHN into salicylate. In contrast, ADPWH_Nag and ADPWH_Nah remained silent in the absence of DHN. The negative control ADPWH_1274 did not respond to DHN (Fig. 4).

**Table 2:** Strains and plasmids used in this study.

Bacteria and Plasmids	Description	Reference
***Acinetobacter baylyi*** ** strains**		
ADP1(BD413)	Wild type	[62]
ADPWH_*lux*	A salicylate biosensor. The promoterless *luxCDABE* from pSB417 wereinserted between the *salA* and *salR* genes in the chromosome of ADP1	[28]
ADPWH_1274	ADPWH_*lux* containing the plasmid pWH1274; Amp^R^	This study
ADPWH_Nag	ADPWH_*lux* containing the plasmid pWH1274 inserted with a positive genecluster capable of transforming 1,2-dihydroxynaphalene to salicylate. Thetinserted gene cluster was from the ^13^C-DNA fraction.	This study
ADPWH_Nah	ADPWH_*lux* containing plasmid pWH1274 with *nahFCQED* from pDTG1in *Pseudomonas putida* NCIB9816. The gene cluster was inserted intoBamHI site of pWH1274	This study
***Pseudomonas*** ** strains**		
*P. putida* NCIB9816	Naphthalene degrader	[29]
*P. putida* UWC1	Spontaneous rifampicin-resistant mutant of *P. putida* KT2440	[30]
*P. putida* WH1	Naphthalene degrader	This study
*P. fluorescens* WH2	Naphthalene degrader	This study
*P. putida* WH3	Naphthalene degrader	This study
**Plasmids**		
pWH1274	*E. coli* - *A. baylyi* shuttle plasmid; Amp^R^, Tet^R^	[37]
pGEM-T	Commercial TA cloning vector	Promega
pDTG1	*nahFCQED* source plasmid, from *Pseudomonas putida* NCIB9816	[31]
pWH_NagFCQED	An unknown *nagFCQED* gene cluster cloned into BamHI site of pWH1274	This study
pWH_NahFCQED	*nahFCQED* cloned into the BamHI site of pWH1274	This study

**Figure 5:**
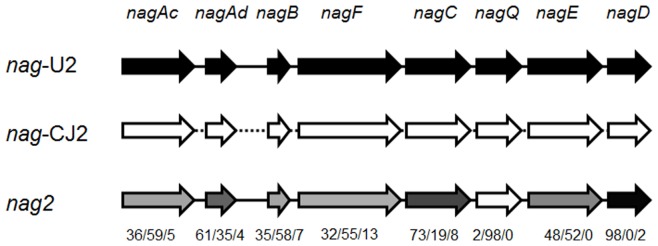
Structure of the *nag* operons revealed by BGT and pyrosequencing. The *nag2* operon was of a mosaic-type pattern. The gray scale of individual genes within the *nag2* operon indicates % similarity to *nag*-U2 (black) or *nag*-CJ2 (white). The numbers beneath the *nag*2 operon indicates the % similarity to *nag*-U2/*nag*-CJ2/neither.

The full-length sequence of the inserted gene cluster *nagFCQED* and NDO PCR products (containing *nagAc* and *nagAd*) were consistent with the pyrosequencing reads of *nag2* (Fig. 5 and S6), confirming that this functional cluster *nag2* represented a new operon. A single pyrosequencing read in the ^13^C-DNA fraction linked *nag*B and *nag*F (Fig. S7) in the *nag*2 operon suggesting that a complete *nag*2 operon for naphthalene biodegradation was present in the groundwater. Interestingly, comparisons of the individual gene sequences *nagAc, Ad, B, C, D, E, F, Q* in the *nag2* operon showed a mosaic-pattern. *nagD* and *nagQ* were similar to their counterpart genes in *Ralstonia* sp. U2 and *P. naphthalenivorans* CJ2 respectively, whereas *nagAc, nagAd, nagB nagF*, *C,* and *E,* were partially similar to both *Ralstonia* sp. U2 and *P. naphthalenivorans* CJ2 (Fig. 5 and S6). A few unassigned DNA sequences were also observed in *nagFCQED* and *nagAc, nagAd* and *nagB,* which may have been due to point mutations (Fig. 5 and S6).

## Discussion

Although metagenomic analyses have a great potential to identify putative novel genes and gene clusters, a better understanding of microbial community function requires functional analyses that can be linked to community members. In this report we integrated SIP, metagenomics and a biosensor-based gene transducer technique to investigate the role of microbial naphthalene biodegradation and the corresponding functional gene clusters in contaminated groundwater.

### BGT as a New Strategy For Function-based Metagenomics

A number of screening approaches have been developed previously for function-based metagenomics, including colony pigment identification [38], antibiotic and enzymatic activity selection [39], [40], substrate-induced gene expression [41] and also *in-vitro* compartmentalization screening [42]. However, these strategies are difficult to apply for screens of metabolic pathways involving small molecules. A BGT system could provide an alternative and sensitive strategy to screen for gene clusters whose expression produce small molecules that activate biosensors (Fig. 1B) [43], [44], [45].

Although metagenomic analysis of ^13^C enriched DNA had identified the *nag2* operon, its functionality was unknown. Moreover, it was not clear whether the *nag2* cluster was from the same operon or an artifact due to inappropriate assembly of short (<530 bp) pyrosequencing reads. Therefore the putative gene cluster was amplified directly from the ^13^C-enriched DNA and cloned into the BGT system to confirm its function in naphthalene metabolism. Salicylate is a central metabolite found in all known aerobic naphthalene biodegradation pathways, including Gram-positive and negative- bacteria [35], [36], [46], [47], [48], [49]. Hence, the salicylate biosensor *A. baylyi* ADPWH_*lux* [28] was employed to ‘light up’ in response to expression of the 4973-bp *nagFCQED* gene cluster that converted DHN into salicylate (Fig. 1B and Fig. 4). Although in this instance the product was amplified by PCR and cloned directly into the expression system, this same approach could, in theory, be used to screen functional genes from a DNA clone library. A variety of biosensors [28], [50], [51], [52], [53] can be potentially tailored to search and screen for genes of interest.

Although functional gene screening using BGT is a powerful approach for gene discovery in uncultured fractions, there are a number of limitations. For screening of environmental samples, BGT would be performed better if it is combined with SIP, thus limits BGT to the study of carbon metabolism. In addition, BGT will be strongly influenced by the maximum size of fragment that is taken up by the host and library size. It would also be difficult to apply BGT to screen functional genes that are not clustered together.

### Multiple Naphthalene Degradation Operons Co-existed in the Same Groundwater and Biased Horizontal Gene Transfer (HGT) Occurred in the Microbial Community

In the groundwater microbial community, at least five naphthalene degradation operons were discovered: *P. putida* NCBI9816-type *nah* (in *P. putida* WH1 and WH3), *P. stutzeri* AN10-type *nah* (in *P. fluorescens* WH2), *Ralstonia* sp. U2-type *nag*, *Polaromonas naphthalenivorans* CJ2-type *nag* and mosaic-pattern *nag2*. The *nag2, nag*-CJ2 and *nag*-U2 operons were present in active microbial community (the ^13^C-DNA enriched fraction), whilst the *nah* operon was not active *in-situ*. The DNA sequence of the *nag2* cluster cloned by PCR and expressed by BGT is consistent with the assembled pyrosequencing sequence (Fig. S6), precluding the possibility that the new discovered *na*g*2* cluster was due to pyrosequencing bias, PCR or DNA assembly artefacts.

In the ^13^C-DNA fraction, *Acidovorax* sp. WH (55%) was the dominant species while *nag2* (64.3%) was the most abundant functional gene cluster for naphthalene degradation, suggesting that *Acidovorax* sp. WH may be associated with *nag*2. In our previous report, we have already proven that *Acidovorax* sp. WH was indeed a key degraders in-situ by using Raman-FISH (fluorescence in-situ hybridization) [26]. It is possible, however, that the correlation between the copies of genes and the abundance of species is not obvious, since gene copy number may vary (e.g. multi-copy gene in chromosome or gene located on plasmids), and high gene expression (at mRNA and protein levels) would made low-copy genes a major function contributor. Ultimately, single cell genomics would provide a confirmation to link the functional genes and their associated species.

Although the *nah* and *nag* operons were present in the same microbial community and the *nah* operons could be disseminated through conjugative plasmids, the *nah* and *nag* operons were preferentially associated with different bacterial groups: *Pseudomonadales* and *Burkholderiales* orders respectively. The sequence of the *nag*2 gene cluster in *Acidovorax* sp. WH shares high similarity to the closely related *Burkholderiales* (*Ralstonia* sp. U2 and *P. naphthalenivorans* CJ2) rather than the distantly-related *Pseudomonadales*. The *nah* operons only disseminated within *Pseudomonadales*, whilst the *nag* operons transferred between the *Burkholderiales*. This suggests that a biased horizontal gene transfer (HGT) [54] could have occured, whereby bacteria preferentially exchange DNA with closely related species.

Sequence- and function-based metagenomics are like two sides of a coin. Sequence-based metagenomics ‘reads’ the life code of a microbial community and function metagenomics aims to ‘understand’ the meaning of the DNA code. One challenge for a function-based metagenomics approach is to develop screening strategies to identify the desired clones. BGT, as a part of the SMB toolbox, is able to characterize genes involving metabolism of molecules which contribute little distinguishable activity or phenotypes to the host clones. An additional advantage of BGT is that a biosensor-based transducer can indicate gene expression activity in a quantitative manner. Hence, BGT potentially enable screening and fine-tuning ‘trapped’ gene expression after mutagenesis (e.g. directed evolution). In this way, after the screening of user-customised ‘*biobricks*’, the discovered *biobricks* can be further optimised for gene expression in the same BGT system. The SMB toolbox will shed light on the ‘hidden’ world of uncultured microorganisms and reveal microbial genetics that culture-dependent approaches and conventional functional metagenomics cannot offer.

## Materials and Methods

### Bacteria Strains, Plasmids, Culture Media and Chemicals

The bacterial strains and plasmids used in this study are listed in Table 2. *Pseudomonas putida* NCIB9816 was obtained from Professor Peter Williams’s laboratory (University of Wales, Bangor, UK) and *Pseudomonas putida* UWC1 is a spontaneous rifampicin resistant mutant of *Pseudomonas putida* KT2440 (a gift from Dr. Andrew Lilly, CEH-Oxford, UK). Luria-Bertani broth (LB) and agar (LBA) (Fisher Scientific) or a minimum medium (MM) were used for bacterial cultivation. One litre MM contains 2.5 g Na_2_HPO_4_, 2.5 g KH_2_PO_4_, 1.0 g NH_4_Cl, 0.1 g MgSO_4_• 7H_2_O, 10 µl CaCl_2_ solution (745 g/l), 10 µl FeSO_4_ solution (256 g/l) and 1 ml Bauchop & Elsden solution [55]. MM-succinate (MMS) was prepared by adding 20 mM succinate (final concentration) to MM. A final concentration of 300 µg/ml ampicillin (Amp) or 10 µg/ml kanamycin (Km) for *A. baylyi* ADP1 and its derivatives were applied when required. *A. baylyi* strains were grown at 30°C.

All chemicals were purchased from Sigma-Aldrich Co., UK and were of analytical-grade unless otherwise stated. The uniformly ^13^C-labelled naphthalene (>99% ^13^C) was purchased from Isotec Sigma-Aldrich (OH). Naphthalene and DHN were dissolved in dimethyl sulfoxide (DMSO) to make 3.8 mM and 100 mM stock solutions respectively, and sodium salicylate was dissolved in distilled water to make 10 mM stock solution. All of the stock solutions were filter-sterilised by passing through 0.22 µm syringe filters (Millipore Inc.).

### Contaminated Site Characterisation and Groundwater Sampling

The contaminated site, from which the groundwater for this study was acquired, is located in Southwest England and has been described previously [26], [27]. Briefly, the groundwater and soil were contaminated by complex polycyclic aromatic hydrocarbons (PAHs). A sequential permeable reactive barrier (PRB) was installed to aerobically treat the mixed contaminants within the groundwater. The main contaminant in the plume across the site is naphthalene with an average concentration of 3.8 µM. The groundwater temperature was consistently 14±2°C [27]. The groundwater was sampled from the inlet of the PRB where contaminated groundwater converged before entering the PRB. The samples were collected in pre-sterilised bottles and were immediately sealed and stored at 4°C in the dark for further analysis.

### Cultured Fractions: Isolation of Naphthalene Degraders

Naphthalene degraders were isolated by spreading 200 µl groundwater on MM plates, which contains no carbon source. Naphthalene crystals were added on the lid of the inverted Petri dishes which vaporised to supply a volatile carbon source to the MM. Negative controls were performed by plating the groundwater on MM plates only. Plates were incubated at 14°C for 1 week to allow colonies to develop. Each experiment was carried out in triplicate.

### Bacterial Conjugation

Conjugation was used to identify the location of the degradation genes. The plasmid-free *P. putida* UWC1 is rifampicin resistant, it cannot utilise naphthalene and was used as the recipient strain for the conjugation experiments. The isolated naphthalene degraders, *P. putida* (WH1 and WH3) and *P. fluorescens* (WH2) which were unable to grow on LB medium containing 100 µg/ml rifampicin, were used as the donor strains. *P. putida* NCIB 9816 was used as a positive control donor. Membrane conjugation mating was performed between each naphthalene degrader and *P. putida* UWC1. Overnight LB cultures of WH1, 2 3, NCIB9816 and UWC1 (5 ml each) were washed with phosphate buffered saline (PBS) three times. The donor cultures (0.5 ml each) were mixed with 0.5 ml UWC1 separately. Each mixture was added onto a cellulose nitrate membrane filter (25 mm diameter, 0.2 µm pore size, Millipore Inc.). The filters, with the bacteria side up, were set on a LBA plate and incubated at 28°C for 24 h. Cells were removed from the filters by vortex-mixing and re-suspended in 10 ml PBS, diluted and plated on MM agar plates, which were supplemented with 100 µg/ml rifampicin and naphthalene as the sole carbon source. The plates were incubated at 28°C for 48 h. All experiments were carried out in triplicate.

### Large Plasmid Isolations

The isolated naphthalene degraders, *P. putida* (WH1 and WH3), *P. fluorescens* (WH2) and *P. putida* NCIB9816, were inoculated in MM supplemented with 2 mM naphthalene. After growing at 28°C with 150 rpm shaking for 3 days, cells were harvested by centrifugation at 3500 rpm for 10 min. Cells were washed three times with PBS and then loaded in a bench-top Nucleoplex BAC Automated DNA Purification System (Nucleoplex, T1000, Tepnel Co., Manchester, UK). The plasmid extraction was carried out using Nucleoplex BAC DNA kit (Tepnel Co., Manchester, UK). After extraction, the plasmids were digested with EcoRI and electrophoresed in a 0.8% agarose gel to view the restriction fragment patterns.

### Groundwater Incubated at Different Temperature and Naphthalene Concentrations

Two hundred and fifty millilitres of groundwater were incubated in the dark at 14°C (groundwater temperature in the field) and or 20°C with final concentrations of naphthalene of 0, 3.8 and 30 µM. Three replicates were carried out for each treatment. After 168-h incubation, total DNA was extracted and used as the template for PCR-DGGE analyses of 16SrRNA genes.

### In-situ ^13^C-naphthalene Enrichment and Measurement of Naphthalene Degradation

An uniformly ^13^C-labelled naphthalene stock solution was added to 250 ml groundwater at a final concentration of 3.8 µM and incubated in the dark at 14°C. Two replicates were performed. As an intermediate metabolite of naphthalene catabolism, accumulation of salicylate in the system was chosen as an indicator of naphthalene degradation as demonstrated in previous studies [26]. Two microlitre aliquots were taken to monitor the naphthalene degradation at the following time points: 0, 24, 48, 54, 72, 96, 120, 144 and 168 h. Detection of salicylate concentration was implemented using the well-characterised biosensor ADPWH_*lux* [28] which expresses bioluminescence in the presence of salicylate.

### Total Nucleic Acids Extraction

Two hundred and fifty millilitres of the labeled groundwater sample was passed through a 47 mm-diameter Sterifil aseptic system filter with a 0.22 µm pore size (Millipore Inc.). The filter was subsequently packed into a BIO-101 tube (Q-biogene) and then 1 ml of DNA extraction buffer (100 mM Tris-Cl (pH 8.0), 100 mM sodium EDTA (pH 8.0), 100 mM phosphate buffer (pH 8.0), 1.5 M NaCl, 1% cetyl-tri-methyl ammonium bromide) was added to the tube, which was subsequently incubated in a water bath at 65°C for 30 min. The sample was subjected to agitation in a FastPrep FP120 bead-beating system (Bio-101, Vista, CA) for 30 s at a speed of 5.5 m/s. The aqueous phase was separated by centrifugation (14000 rpm, 5 min) and then the proteins were precipitated by adding an equal volume of chloroform: isoamylalcohol (24∶1 v/v) and removed by centrifugation (14000 rpm, 5 min). Thereafter the nucleic acids were isolated by precipitation with isopropanol for 2 h, centrifuged at 14,000 rpm for 10 min, washed in 70% (v/v) ethanol, air dried, re-suspended in 50 µl RNase-free water (Invitrogen) and stored at −20°C for later analysis.

### Stable Isotope Probing Fractionation

Separation and recovery of the ^13^C-labelled and unlabeled background community DNA was carried out following a modified fractionation-based approach, through which caesium chloride (CsCl) solution was collected drop-wise from the bottom of the ultra-centrifuge tube [56]. Briefly, DNA (1 µg) was mixed with the CsCl gradient buffer, giving a total volume of 1.2 ml. This was combined with 4.8 ml of 7.163 M CsCl solution in a 15-ml screw-cap tube, inverted gently, transferred into an ultracentrifuge tube (Beckman), sealed, balanced and subjected to ultra-centrifugation (Optima L-80 XP Ultracentrifuge, Beckman Coulter) at 44,100 rpm (∼177,000 g, VTi65.2 rotor, Beckman) for 40 h. Afterwards, DNA was retrieved by gradient fractionation, yielding approximately 12 × 425 µl fractions collected from the bottom of the centrifuge tube with sterile deionised water injected from the top using a low-flow peristaltic pump (Watson Marlow Ltd.). The density of each fraction was measured using a digital refractometer as described previously [56]. Then the DNA from all fractions were precipitated by glycogen and PEG solution for 2 h, washed with 70% ethanol, air-dried and dissolved in 30 µl DNase-free water. Control experiments with ^12^C-napthalene were set up and subsequent centrifugation, fractionation and DNA precipitation were also carried out.

### General DNA Manipulation

Established methods were used for the DNA purification, digestion with restriction endonucleases, ligation and agarose gel electrophoresis (1% unless otherwise specified) [57]. All used restriction endonucleases and modification enzymes were purchased from New England Biolabs. Ligations were performed using Fast-Link DNA Ligation Kits (EPICENTRE) following the manufacturer’s instructions. Plasmid isolation from the *Acinetobacter* (50 ml overnight culture) was performed using the QIAprep Spin Miniprep Kit (QIAGEN). DNA fragments were purified using either a QIAquick PCR Purification Kit or a QIAquick Gel Extraction Kit (QIAGEN) as appropriate. Primers (Table 1) for PCR and sequencing were purchased from Eurofins MWG Operon. PCR amplifications were carried out in a 50 µl reaction containing 1× reaction PCR buffer (Fermentas Co. UK), 200 µM of each deoxynucleotide triphosphate (Bioline), 0.5 µM of each primer, 1–2 unit DreamTaq DNA polymerase (Fermentas Co. UK) and 50 ng template DNA. PCR was accomplished following the manufacturer’s instructions (Fermentas Co. UK).

### PCR Amplification and DGGE Analysis of 16S rRNA Genes

The ^12^C- and ^13^C-DNA fractions were separately used as templates for PCR amplification of NDO genes using degenerate primers of *Comamonas*-type and *Pseudomonas*-type NDO (Table 1). The PCR products were cloned into the pGEM-T vector as the manufacturer’s instruction. Ten pGEM-T clones bearing NDO genes were chosen randomly, purified and sequenced. The purified ^12^C- and ^13^C-DNA from each fraction was amplified using the 16S rRNA gene primer pairs GC338F and 530 R, and the GC-clamped products were loaded on a 10% (w/v) polyacrylamide gel with a 30–60% urea/formamide denaturing gradient [58]. The denaturing gradient gel was cast and then run using the Ingeny PhorU2 system at 60°C for 16 h. Gels were stained with SYBR gold nucleic acid stain and visualised by a VersaDoc Imaging system (MP4000, Bio-Rad Laboratories). Bands of interest were excised from the gel, re-amplified and sequenced to provide phylogenetic information.

### Nucleotide Sequencing and Computational Analysis

The ^13^C-DNA fraction was amplified using a REPLI-g Mini Kit (Qiagen, UK) according to the manufacturer’s instruction prior to pyrosequencing. Metagenomic sequencing of total ^13^C and ^12^C-DNA was carried out using a 454 sequencing platform (Genome Sequencer FLX system, Roche Applied Science). Assembly of the sequence data was performed through *GS De Novo Assembler* (Roche), (also called *Newbler Assembler*). Taxonomic analysis based on 16S rRNA genes was accomplished using a recently developed software Parallel-META (http://www.computationalbioenergy.org/parallel-meta.html) and compared to the SILVA database [59] to investigate the relative abundance of different species present in the ^13^C-incorporated community.

PCR products were sequenced using a 48-capillary 3730 DNA Analyzer (Applied Biosystems) and the sequence data was analysed using BioEdit (Tom Hall, North Carolina State University). The insertion of the DHN degradation gene cluster in pWH1274 was confirmed by sequencing and further alignment using the primers pWH1274_P12 and pWH1A1_1_P4R, and the sequence of this whole gene cluster was determined by assembly of sequence data obtained using the primers Nag_F, NagSeq1_F, NagSeq2_F, NagSeq3_F1, NagSeq3_F2, NagSeq4_F, NagSeq5_F and NagD_R. Homology searches were performed by BLAST available at the National Center for Biotechnology Information website (http://blast.ncbi.nlm.nih.gov/Blast.cgi).

### Cloning of a Putative DHN Degradation Gene Cluster

A putative DHN degradation gene cluster was identified by homology alignment to the known sequences in the NCBI database and obtained by PCR using the primers NagF1_For and NagD_Rev with 1 µl ^13^C-DNA as the template. The products were then ligated into the BamHI site of pWH1274, and the mixture transferred into ADPWH_*lux* by electroporation. The transformants were incubated in MM containing 50 µM DHN with bioluminescence as a selection marker for the positive clones.

### Kinetic Detection of Bioluminescence

The bioluminescence signal of the *Acinetobacter* strains was detected using a Synergy 2 multimode microplate reader (BioTek Instruments, Inc., USA) as described previously [50], [52]. Cells were cultured for 18 h at 30°C in MMS (with appropriate antibiotics) and then 20 µl of the cells were added into 180 µl fresh MMS with the inducer as appropriate. Sodium salicylate 50 µM was used as an inducer for ADPWH_*lux* to characterise the biosensor, 50 µM DHN for the transformants, and 50 µM DMSO as a negative control. The cells were transferred into the wells of a black clear-bottomed 96-well microplate (Fisher Scientific, UK) and incubated for 200 min at 30°C with 4 replicates for each treatment. During the incubation, the bioluminescence intensity and the optical density (OD_600_) were measured every 10 min. The relative bioluminescence was obtained by dividing the bioluminescence intensity by cell density for each well.

### Nucleotide Sequence Accession Number

The DNA sequences have been submitted to NCBI GenBank. The accession number of the *E. coli*-*Acinetobacter* shuttle plasmid pWH1274 is JN381160. The accession number for the *nag2* gene cluster is JN563842. The 16S rRNA genes of the isolated naphthalene degraders, *P. fluorescens* WH2, *P. putida* WH1 and WH3, were previously documented and the accession numbers are EF413073, EF413072, and EF413074 respectively.

## Supporting Information

Figure S1 **Restriction enzyme digests of plasmids.** Plasmids were extracted from *P. putida* WH1, *P. fluorescens* WH2, *P. putida* WH3 and *P. putida* NCIB9816 and digested by EcoRI. Samples were run on a 0.8% agarose gel.
(PDF)

Figure S2 **The effect of temperature and naphthalene concentration on the structure of the microbial community determined by 16S rRNA DGGE analysis.**
(PDF)

Figure S3 **Salicylate (an intermediate metabolite) accumulation in groundwater indicating naphthalene (3.8 µM) degradation.** Salicylate detection was performed using the salicylate biosensor ADPWH_*lux* which showed bioluminescence in the presence of salicylate. Naphthalene catabolism releasing salicylate was evident after 120 h incubation. Results are the mean +/− SD of 4 replicate measurements.
(PDF)

Figure S4 **16S-rRNA DGGE analysis of the microbial communities in ^13^C- and ^12^C-enriched DNA.** Two duplicate experiments (1 & 2) were performed. F stands for different fractions after SIP separation. The most intense bands B, present in the ^13^C-DNA but not in the ^12^C-DNA (labelled B1, B2 and B3), were excised, re-amplified, and sequenced. The result revealed its affiliation with *Acidovorax* sp. and was designated as *Acidovorax* sp. WH.
(PDF)

Figure S5 **Phylogenetic tree of some classified 16S rRNA sequences in the ^12^C- and ^13^C-DNA fractions.** Species relative abundance of the total 16S-rRNA reads is shown as %.
(PDF)

Figure S6 **Multi-sequence alignment of the** ***nag*** **operons, including** ***nag2*** **,** ***nag*** **-U2 and** ***nag-*** **CJ2.** Bases which were not the same between *nag2*, *nag*-U2 and *nag-*CJ2 are colored. Grey highlight represents homologous bases between *nag2* and *nag*-U2, but different from *nag*-CJ2; Red highlight represents homologous bases between *nag2* and *nag*-CJ2, but different from *nag-*U2; Blue highlight represents bases of *nag2* with no homologies with either *nag*-CJ2 or *nag*-U2. The DNA sequence of *nagFCQED* is from the plasmid pWH_NagFCQED. Bases from single 454 sequencing read are underlined and marked alongside each gene. The numbers on the bottom right are the codes of individual 454 sequence reads.
(PDF)

Figure S7 A single pyrosequencing read in the ^13^C-DNA fraction links *nag*B and *nag*F in nag2 operon.
(PDF)

Table S1 Homology analysis of nag2 and nag-CJ2/nag-U2.
(XLSX)
