# Supplementary material for: A Culture-Independent Approach to Unravel Uncultured Bacteria and Functional Genes in a Complex Microbial Community
Source: PLoS One. 2012 Oct 17;7(10):e47530. doi: 10.1371/journal.pone.0047530 (PMC3474725; doi:10.1371/journal.pone.0047530)
Supplement: Figure S1 — Restriction enzyme digests of plasmids. Plasmids were extracted from P. putida WH1, P. fluorescens WH2, P. putida WH3 and P. putida NCIB9816 and digested by EcoRI. Samples were run on a 0.8% agarose gel. (PDF) [file pone.0047530.s001.pdf]

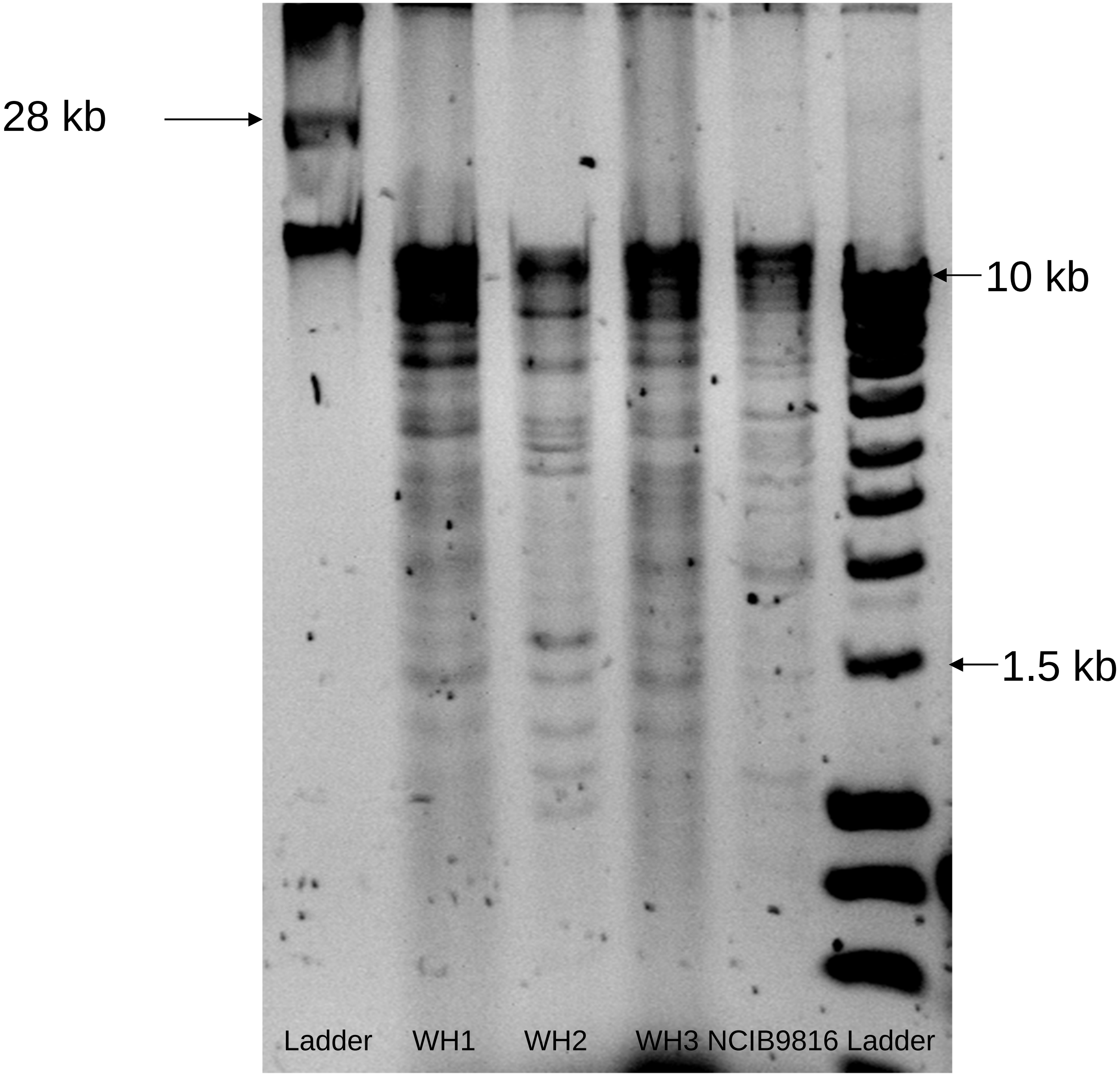

Figure S1. Restriction enzyme digests of plasmids. Plasmids were extracted from *P. putida* WH1, *P. fluorescens* WH2, *P. putida* WH3 and *P. putida* NCIB9816 and digested by EcoRI. Samples were run on a 0.8% agarose gel.
