# Supplementary material for: A Culture-Independent Approach to Unravel Uncultured Bacteria and Functional Genes in a Complex Microbial Community
Source: PLoS One. 2012 Oct 17;7(10):e47530. doi: 10.1371/journal.pone.0047530 (PMC3474725; doi:10.1371/journal.pone.0047530)
Supplement: Figure S2 — The effect of temperature and naphthalene concentration on the structure of the microbial community determined by 16S rRNA DGGE analysis. (PDF) [file pone.0047530.s002.pdf]

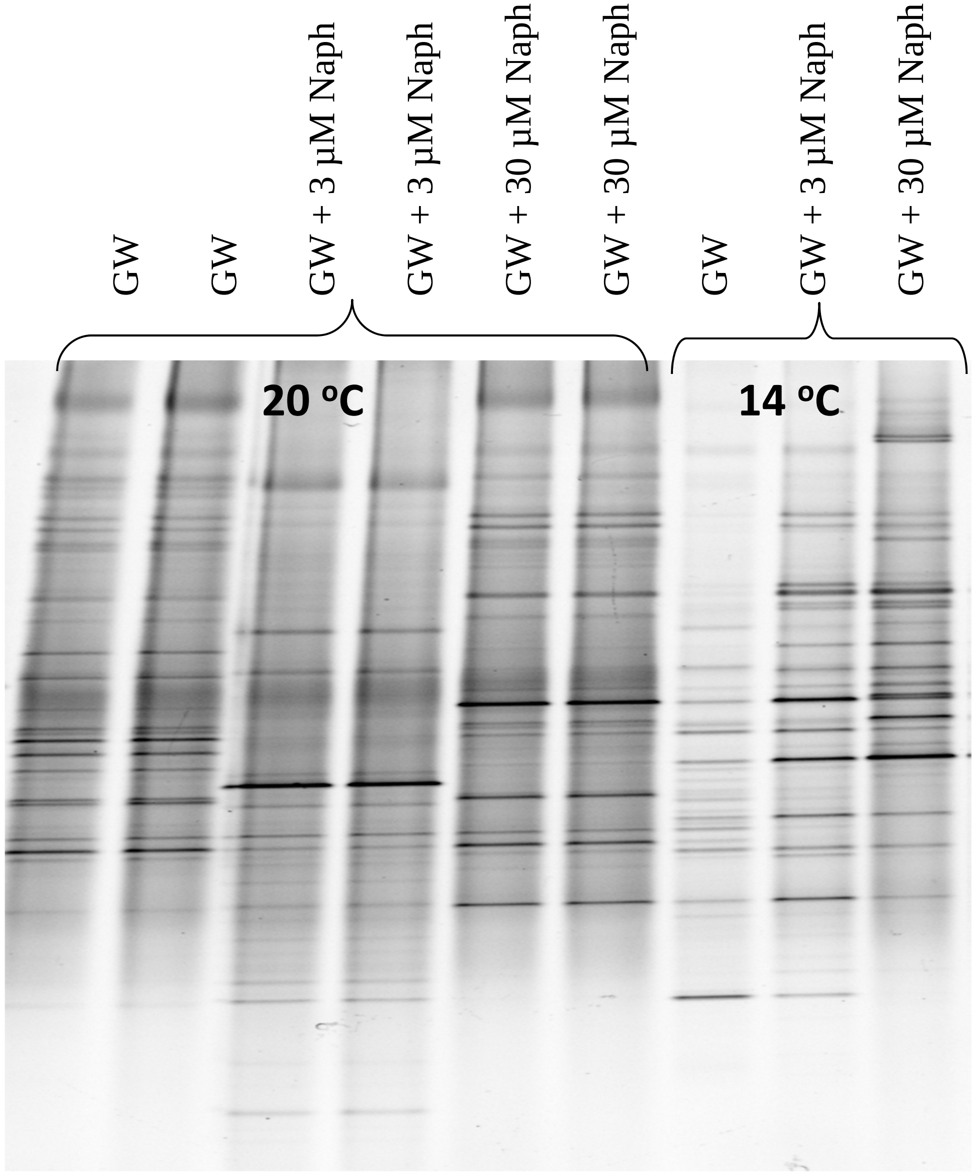

Figure S2. The effect of temperature and naphthalene concentration on the structure of the microbial community determined by 16S rRNA DGGE analysis.
