# Supplementary material for: A Culture-Independent Approach to Unravel Uncultured Bacteria and Functional Genes in a Complex Microbial Community
Source: PLoS One. 2012 Oct 17;7(10):e47530. doi: 10.1371/journal.pone.0047530 (PMC3474725; doi:10.1371/journal.pone.0047530)
Supplement: Figure S3 — Salicylate (an intermediate metabolite) accumulation in groundwater indicating naphthalene (3.8 µM) degradation. Salicylate detection was performed using the salicylate biosensor ADPWH_lux which showed bioluminescence in the presence of salicylate. Naphthalene catabolism releasing salicylate was evident after 120 h incubation. Results are the mean +/− SD of 4 replicate measurements. (PDF) [file pone.0047530.s003.pdf]

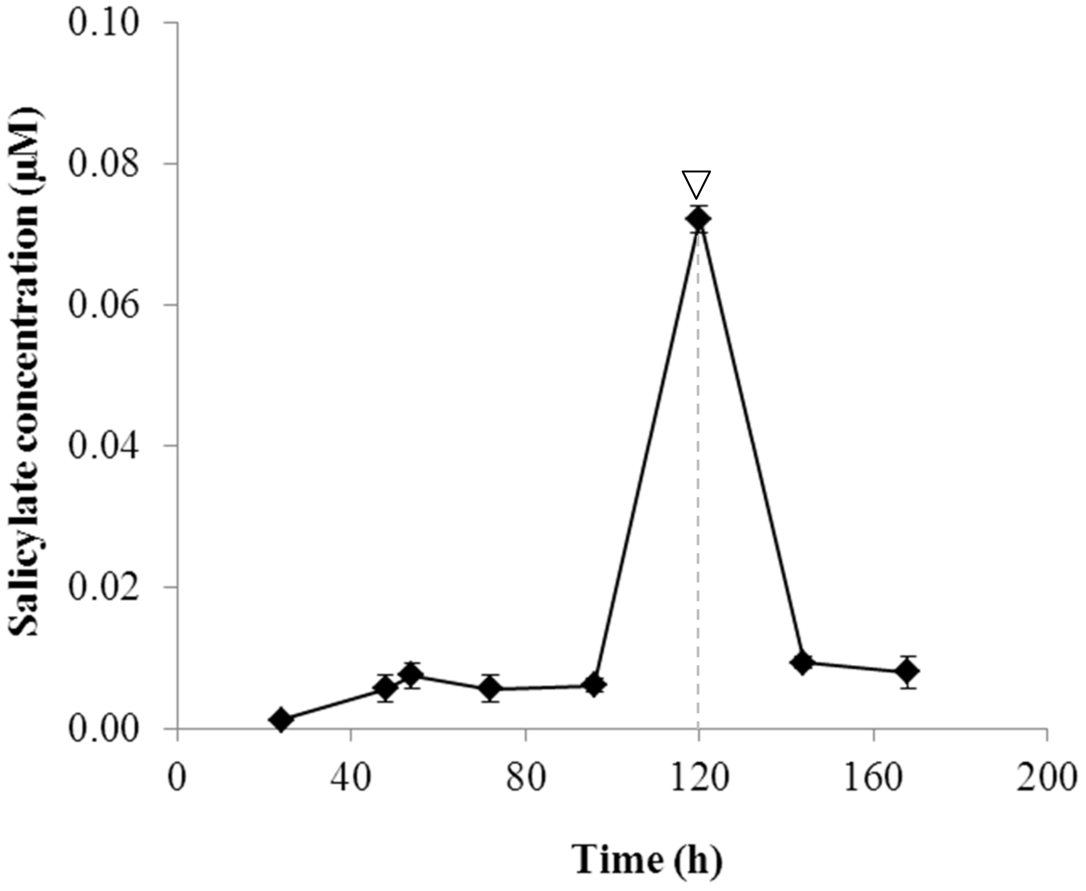

Figure S3. Salicylate (an intermediate metabolite) accumulation in groundwater indicating naphthalene (3.8 μM) degradation. Salicylate detection was performed using the salicylate biosensor ADPWH_*lux* which showed bioluminescence in the presence of salicylate. Naphthalene catabolism releasing salicylate was evident after 120 h incubation. Results are the mean +/- SD of 4 replicate measurements.
