# Supplementary material for: A Culture-Independent Approach to Unravel Uncultured Bacteria and Functional Genes in a Complex Microbial Community
Source: PLoS One. 2012 Oct 17;7(10):e47530. doi: 10.1371/journal.pone.0047530 (PMC3474725; doi:10.1371/journal.pone.0047530)
Supplement: Figure S4 — 16S-rRNA DGGE analysis of the microbial communities in 13C- and 12C-enriched DNA. Two duplicate experiments (1 & 2) were performed. F stands for different fractions after SIP separation. The most intense bands B, present in the 13C-DNA but not in the 12C-DNA (labelled B1, B2 and B3), were excised, re-amplified, and sequenced. The result revealed its affiliation with Acidovorax sp. and was designated as Acidovorax sp. WH. (PDF) [file pone.0047530.s004.pdf]

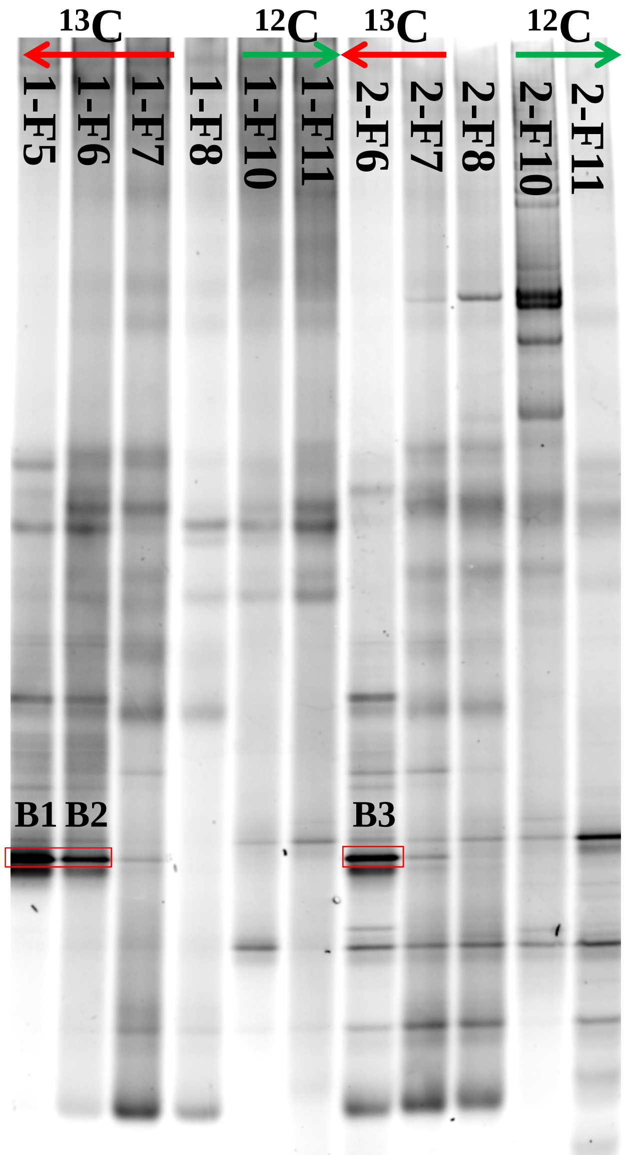

Figure S4. 16S-rRNA DGGE analysis of the microbial communities in ^{13}C- and ^{12}C-enriched DNA. Two duplicate experiments (1 & 2) were performed. F stands for different fractions after SIP separation. The most intense bands B, present in the ^{13}C-DNA but not in the ^{12}C-DNA (labelled B1, B2 and B3), were excised, re-amplified, and sequenced. The result revealed its affiliation with *Acidovorax* sp. and was designated as *Acidovorax* sp. WH.
