# Supplementary material for: A Culture-Independent Approach to Unravel Uncultured Bacteria and Functional Genes in a Complex Microbial Community
Source: PLoS One. 2012 Oct 17;7(10):e47530. doi: 10.1371/journal.pone.0047530 (PMC3474725; doi:10.1371/journal.pone.0047530)
Supplement: Figure S5 — Phylogenetic tree of some classified 16S rRNA sequences in the 12C- and 13C-DNA fractions. Species relative abundance of the total 16S-rRNA reads is shown as %. (PDF) [file pone.0047530.s005.pdf]

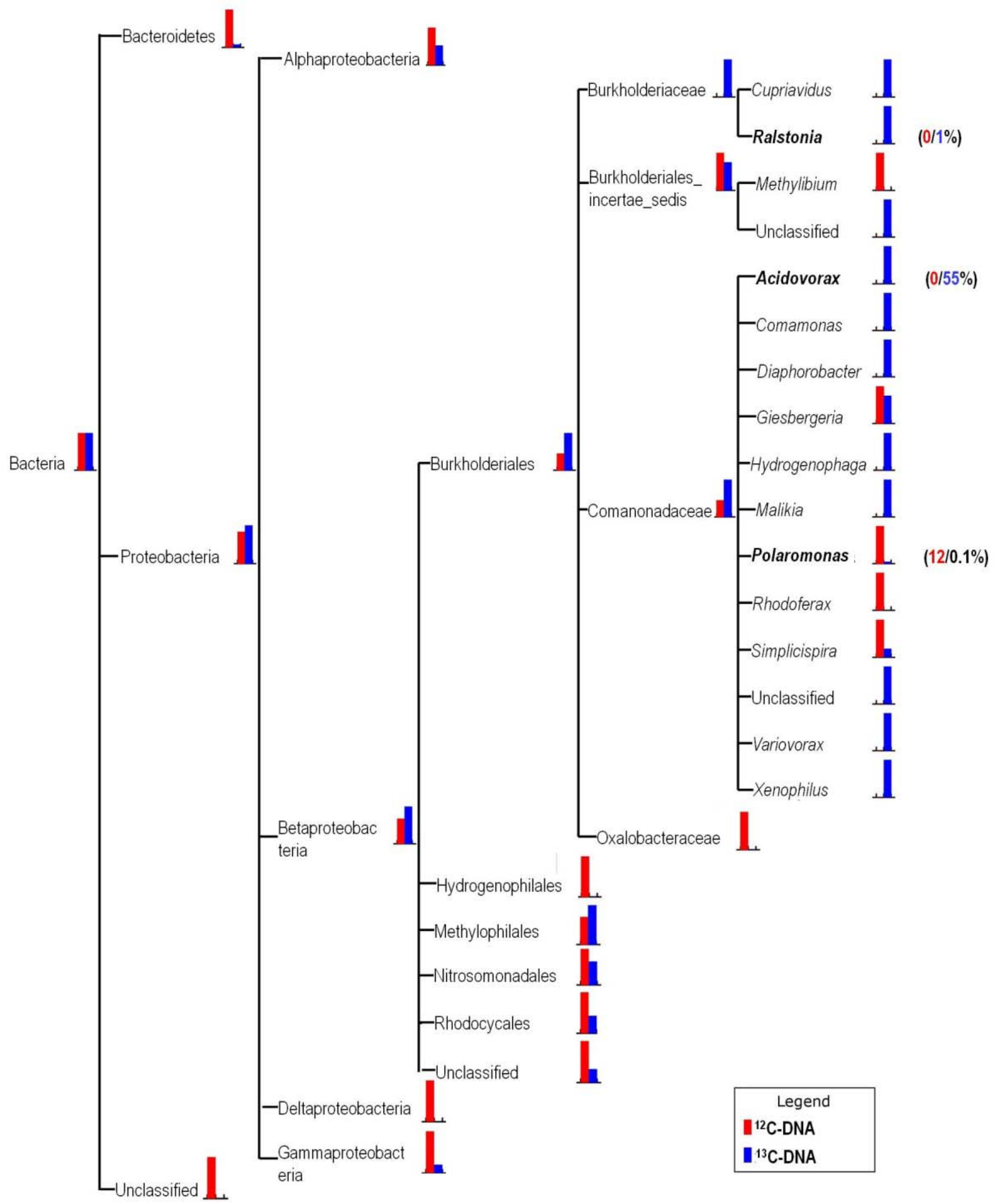

Figure S5. Phylogenetic tree of some classified 16S rRNA sequences in the ^{12}C- and ^{13}C-DNA fractions. Species relative abundance of the total 16S-rRNA reads is shown as %.
