# Supplementary material for: A Culture-Independent Approach to Unravel Uncultured Bacteria and Functional Genes in a Complex Microbial Community
Source: PLoS One. 2012 Oct 17;7(10):e47530. doi: 10.1371/journal.pone.0047530 (PMC3474725; doi:10.1371/journal.pone.0047530)
Supplement: Figure S6 — Multi-sequence alignment of the nag operons, including nag2 , nag -U2 and nag- CJ2. Bases which were not the same between nag2, nag-U2 and nag-CJ2 are colored. Grey highlight represents homologous bases between nag2 and nag-U2, but different from nag-CJ2; Red highlight represents homologous bases between nag2 and nag-CJ2, but different from nag-U2; Blue highlight represents bases of nag2 with no homologies with either nag-CJ2 or nag-U2. The DNA sequence of nagFCQED is from the plasmid pWH_NagFCQED. Bases from single 454 sequencing read are underlined and marked alongside each gene. The numbers on the bottom right are the codes of individual 454 sequence reads. (PDF) [file pone.0047530.s006.pdf]

```
U2   nagAc 319  GGCTCCAACGGCGAACTGCAAAGCGTT-CCCTTTGAAAAAGAGTTGTACGGCGATACGATCAAAAAGAAGTGCCTGGGCTTGAAAGAAGTCCCCCGCATC 418
CJ2  nagAc 319  GGCTCCAACGGTGAACTTCAAAGCGTT-CCCTTTGAAAAAGAATTGTACGGAGATGCGATCAAAAAGAAGTGCTTGGGCTTGAAAGAAGTCCCGCGCGTC 418
GW   nagAc 319  GGCTCCAACGGCGAACTGCAAAGCGTTACCCTTTGAAAAAGAGTTGTACGGCGATGCGATCAAAAAGAAATGCCTGGGCTTGAAAGAAGTCCCCCGCATC 418

U2   nagAc 419  GAAAGCTTTCATGGCTTCATTTACGGTTGTTTTGATGCAGAGGCCCCCACGCTTGTCGATTATCTGGGTGATGCAGCCTGGTACTTAGAACCCATCTTCA 518
CJ2  nagAc 419  GAGAGCTTTCATGGCTTTATTTACGGCTGTTTTGATGCAGAGGCTCCCCCGCTCATGGATTATCTGGGTGATGCAGCCTGGTACCTGGAGCCCATCTTCA 518
GW   nagAc 419  GAAAGCTTTCATGGTTTTATCTACGGCTGTTTTGATGCAGAAGCTCCCCCGCTCATCGATTATCTGGGTGATGCAGCCTGGTACCTGGAGCCCATCTTCA 518

U2   nagAc 519  AGCACTCTGGTGGCCTGGAACTTGTAGGCCCCCCCGGCAAAGTGGTGATCAAGGCCAACTGGAAGGCTCCTGCGGAAAACTTTGTGGGTGACGCGTACCA 618
CJ2  nagAc 519  AGCACTCAGGCGGTCTGGAACTTGTAGGCCCCCCGGGCAAAGTGGTGATCAAGGCCAACTGGAAGGCGCCCTCGGAAAACTTTGTGGGTGACGCATACCA 618
GW   nagAc 519  AGCACTCAGGCGGTCTGGAACTTGTAGGCCCCCCGGGCAAAGTGGTGATCAAGGCCAACTGGAAGGCGCCCTCGGAAAACTTTGTGGGTGACGCATACCA 618

U2   nagAc 619  CGTTGGTTGGACGCACGCATCGTCTTTGCGCTCAGGTCAGTCGATATTTACCCCTCTTGCGGGCAACGCTATGCTTCCACCCGAAGGCGCGGGCTTACAA 718
CJ2  nagAc 619  CGTGGGCTGGACGCACGCATCGTCTTTGCGCTCAGGTCAGTCGATATTTACCCCTCTCGCGGGCAACGCCATGCTGCCGCCCGAAGGCGCGGGCTTGCAA 718
GW   nagAc 619  CGTGGGCTGGACGCACGCATCGTCTTTGCGCTCAGGTCAGTCGATATTTACCCCTCTCGCGGGCAACGCCATGCTGCCGCCCGAAGGCGCGGGCTTGCAA 718

U2   nagAc 719  ATGACCAGCAAGTATGGCAGTGGAATGGGCGTATTGTGGGACGGCTACTCCGGTGTCCACAGTGCTGACCTGGTTCCCGAAATGATGGCATTCGGCGGCG 818
CJ2  nagAc 719  ATGACCAGCAAATACGGCAGCGGCATGGGGGTGTTGTGGGACGCTTATTCCGGCGTTCACAGCGCCGACCTGGTGCCGGACATGATGGCATTCGGCGGCG 818
GW   nagAc 719  ATGACCAGCAAATACGGCAGCGGCATGGGGGTGTTGTGGGACGCTTATTCCGGCGTTCACAGCGCCGACCTGGTGCCGGACATGATGGCATTCGGCGGCG 818

U2   nagAc 819  CAAAACAGGAAAAACTCGCCAAGGAAATCGGCGATGTCCGGGCACGGATTTACCGCAGCCATCTAAACTGCACGGTTTTCCCGAACAACAGCATTTTGAC 918
CJ2  nagAc 819  CAAAACAGGAAAAGCTCGCCAAAGAAATCGGTGATGTTCGGGCGCGGATTTACCGCAGCCATCTGAACTGCACGGTTTTCCCGAACAACAGCATTTTGAC 918
GW   nagAc 819  CAAAACAGGAAAAGCTCGCCAAAGAAATCGGTGATGTTCGGGCGCGGATTTACCGCAGCCATCTGAACTGCACGGTTTTCCCGAACAACAGCATTTTGAC 918

U2   nagAc 919  CTGCTCCGGTGTCTTCAAGGTCTGGAACCCGATCGATGAAAACACGACCGAGGTTTGGACGTATGCCATCGTAGAAAAAGACATGCCTGAGGACTTAAAG 1018
CJ2  nagAc 919  CTGCTCCGGCGTTTTCAAGGTCTGGAACCCGATCGACGAGAACACGACCGAAGTTTGGACGTATGCGATCGTGGAAAAAGACATGCCGGAGGAATTGAAA 1018
GW   nagAc 919  CTGCTCCGGCGTTTTCAAGGTCTGGAACCCGATCGACGAGAACACGACCGAAGTTTGGACGTATGCGATCGTGGAAAAAGACATGCCGGAGGAATTGAAA 1018

U2   nagAc1019  CGTCGCTTGGCTGACGCGGTTCAGCGCACTTTCGGACCAGCAGGATTCTGGGAAAGCGACGACAACGACAACATGGAGACGGAGTCGCAAAATGCCAAGA 1118
CJ2  nagAc1019  ATCCGCTTAGCCGACGCGGTTCAGCGCACGTTCGGACCTGCAGGCTTCTGGGAAAGCGACGACAACGACAACATGGAAACCGAGTCGCAAAATGCCAAGA 1118
GW   nagAc1019  ATCCGCTTAGCCGACGCGGTTCAGCGCACGTTCGGACCTGCAGGCTTCTGGGAAAGCGACGACAACGACAACATGGAGACGGAGTCGCAAAATGCCAAGA 1118

U2   nagAc1119  AATACCAATCCAGCAACAGTGATCTGATTGCCAATTTGGGTTTCGGCAAGGACGTCTACGGCGACGAATGCTATCCGGGCGTCGTTGCCAAATCGGCAAT 1218
CJ2  nagAc1119  AATACCAATCCAGCAACAGCGATCTGCTTGCCAACCTGGGTTTCGGCAAGGACGTCTACGGTGATGAAGTATATCCCGGCGTCGTTGGCAAGTCGTCCAT 1218
GW   nagAc1119  AATACCAATCCAGCAACAGTGATCTGATTGCCAATTTGGGTTTCGGCAAGGACGTCTACGGCGACGAATGCTATCCGGGCGTCGTTGCCAAATCGGCAAT 1218

U2   nagAc1219  CGGCGAAACCAGCTATCGCGGATTCTACCGTGCCTACCAGGCTCACATCAGCAGCTCCAATTGGGCCGAGTTCGAAAACACCTCCCGAAATTGGCACACC 1318
CJ2  nagAc1219  CGGTGAAACCAGCTATCGCGGCTTCTACCGCGCCTACCAAGCGCACATCAGCAGCTCCAATTGGGCCGAGTTCGAAAACAGCTCACGCAGTTGGCATGCC 1318
GW   nagAc1219  CGGCGAAACCAGCTATCGCGGATTCTACCGTGCCTACCAGGCTCACATCAGCAGCTCCAATTGGGCCGAGTTCGAAAACACCTCCCGAAATTGGCACACC 1318

U2   nagAc1319  GAACTCACCAAGACGACTGATCGCTAA 1345
CJ2  nagAc1319  GAACTCACCCAGACCGCCGATCGCTAA 1345
GW   nagAc1319  GAACTCACCAAGACGACTGATCGCTAA 1345
```

──────── GYVI30E01AX3SN

— · — GYVI30E01ERXW9

════════ GYVI30E02FYFOH

```
U2    nagAd 1    ATGATGATCAATACCCAGGAAGACAAGCTGGTCTCCGCGCACGACGCCGAAGAATTTCATCGTTTCTTCGTCGGGCACGACAGCGATCTGCAGCAAGAAG 100
CJ2   nagAd 1    ATGATGATCAACACCCAGGAAGACAAGCTGGTCTCCGCGCACGACGCTGAAGAATTTCATCGCTACTTCATCGGACACGATAGCGCTTTGCTGCAAGAAG 100
GW    nagAd 1    ATGATGATCAATACCCAGGAAGACAAGCTGGTCTCCGCGCACGACGCTGAAGAATTTCATCGTTTCTTCGTCGGGCACGACAGCGATCTGCAGCAAGAAG 100

U2    nagAd 101  TCACCACACTCCTGACCCGAGAAGCACATCTGTTGGACATTCAGGCCTACAACGCCTGGCTTGAACACTGCGTTGCCCCCGAGATCAAATACCAAGTGAT 200
CJ2   nagAd 101  TCAACACGCTCCTAGCCAGGGAAGCACATCTGTTGGACATTCAGGCATACAAGGCTTGGCTTGAACACTGCGTTGCCCCCGAGATCAAATACCAGGTGAT 200
GW    nagAd 101  TCACCACACTCCTGACCCGAGAAGCACATCTGTTGGACATTCAGGCCTACAACGCCTGGCTTGAACACTGCGTTGCCCCCGAGATCAAATACCAAGTGAT 200

U2    nagAd 201  CTCGCGAGAATTTCGGTCCACTTCCGAGCGTCGATACCAACTGAATGATGCGGTGAACATCTACAACGAGAACTATCAGCACCTGAAAGTTCGAGTTGAG 300
CJ2   nagAd 201  CTCGAGAGAACTGCGTTCCACCTCTGAACGCCGGTACAAGCTGAATGACGCAGTGAACATCTACAACGAGAACTATCAGCAACTGAAAGTTCGAGTTGAG 300
GW    nagAd 201  CTCGCGAGAACTTCGCTCCACTTCCGAGCGTCGATACCAACTGAATGATGCGGTGAACATCTACAACGAGAACTATCAGCAACTGAAAGTTCGAGTTGAG 300

U2    nagAd 301  CATCAGATGGACCCGCAGAACTGGGCCAACAGCCCGAAGATCCGTTTCACCCGCTTCGTCACCAATGTCACAGCGGCCAAGGACAAGATCGTACCGGATC 400
CJ2   nagAd 301  CATCAGCTCGACCCGCAGAATTGGGCCAACAGCCCGAAGATGCGTTTCACTCGCTTCGTCACCAATGTCATGGCAGCCAAGGACACGGGGGCACCGGATC 400
GW    nagAd 301  CATCAGCTCGACCCGCAGAATTGGGCCAACAGCCCGAAGATGCGTTTCACTCGCTTCGTCACCAATGTCATGGCAGCCAAGGACACGGGTGCACCGGATC 400

U2    nagAd 401  TGCTGCATGTGCGTTCCAACCTCATTCTCCAC 432
CJ2   nagAd 401  TGCTGCATGTCCGCTCCAACCTCATTCTCCAC 432
GW    nagAd 401  TGCTGCATGTCCGCTCCAACCTCATTCTCCAC 432
```

—— GYVI30E01DH2TJ

```
U2   nagB 476 TTTCTTCCTATGCGGCCGGCGGCGGCGGCTCTTGCTACATTGCCAGCAAGCATGCAGTGCTGGGTATGGTCAAGGCGTTGGCCTACGAATTGGCCCCGCA 575
CJ2  nagB 476 TTTCTTCCTATGCGGTCGGTGGCGGCGGTTCTTGCTACATTGCCAGCAAACATGCGGTGCTGGGCATGGTCAAGGCTTTGGCCTACGAATTGGCTCCGCA 575
GW   nagB 476 TTTCTTCCTATGCGGTCGGTGGCGGCGGTTCTTGCTACATGGCCAGCAAACATGCGGTGCTGGGCATGGTCAAGGCTTTGGCCTACGAATTGGCTCCGCA 575

U2   nagB 576 CATCCGGGTCAATGGCGTTGCGCCAGGTGGTACGGTCACTTCTTTGGCTGGGCCGGCAAGCGCCGGTTTCGACAAAACCAAAATGAAAGACATGCCCGGC 675
CJ2  nagB 576 CATCCGGGTCAATGGCGTTGCGCCAGGTGGCACGGTCACTTCTTTGGCTGGCCCGGCAAGCGCTGGCTTCGACAAAACCAAAATGAAAGACATTCCCGGC 675
GW   nagB 576 CATCCGGGTCAATGGCGTTGCGCCAGGTGGTGCGGTCACTTCTTTGGCTGGCCCGGCAAGCGCTGGCTTCGACAAAACCAAAATGAAAGACATGCCCGGC 675

U2   nagB 676 ATCGATGACATGATCAAGGGCCTGACCCCCTTGGGGTTCGCAGCAAGGCCCGAGGACGTGGTGGCACCGTATCTTTTGCTGGCCTCCCGGGAACAAGGGA 775
CJ2  nagB 676 ATCGATGACATGATCAAAGGCCTGACTCCCCTGGGGTTCGCGGCAAGGCCCGAGGACGTGGTGGCACCGTACCTTTTGCTGGCCTCCCGAGAACAAGGGA 775
GW   nagB 676 ATCGATGACATGATCAAAGGCCTGACTCCCCTGGGGTTCGCGGCAAGGCCCGAGGACGTGGTGGCACCGTATCTTTTGCTGGCCTCCCGGGAACAAGGGA 775

U2   nagB 776 AGTTCATCACTGGCACCGTAATCGGCATTGATGGCGGCATGGCGCTCGGTCGAAAGTGA 834
CJ2  nagB 776 AGTTCATCACTGGCACGGTGATCAGCATTGATGGCGGCATGGCGCTCGGCCGCAAGTGA 834
GW   nagB 776 AGTTCATCACTGGCACCGTAATCGGCATTGATGGCGGCATGGCGCTCGGTCGAAAGTGA 834
```

—— GYVI30E01D06VU

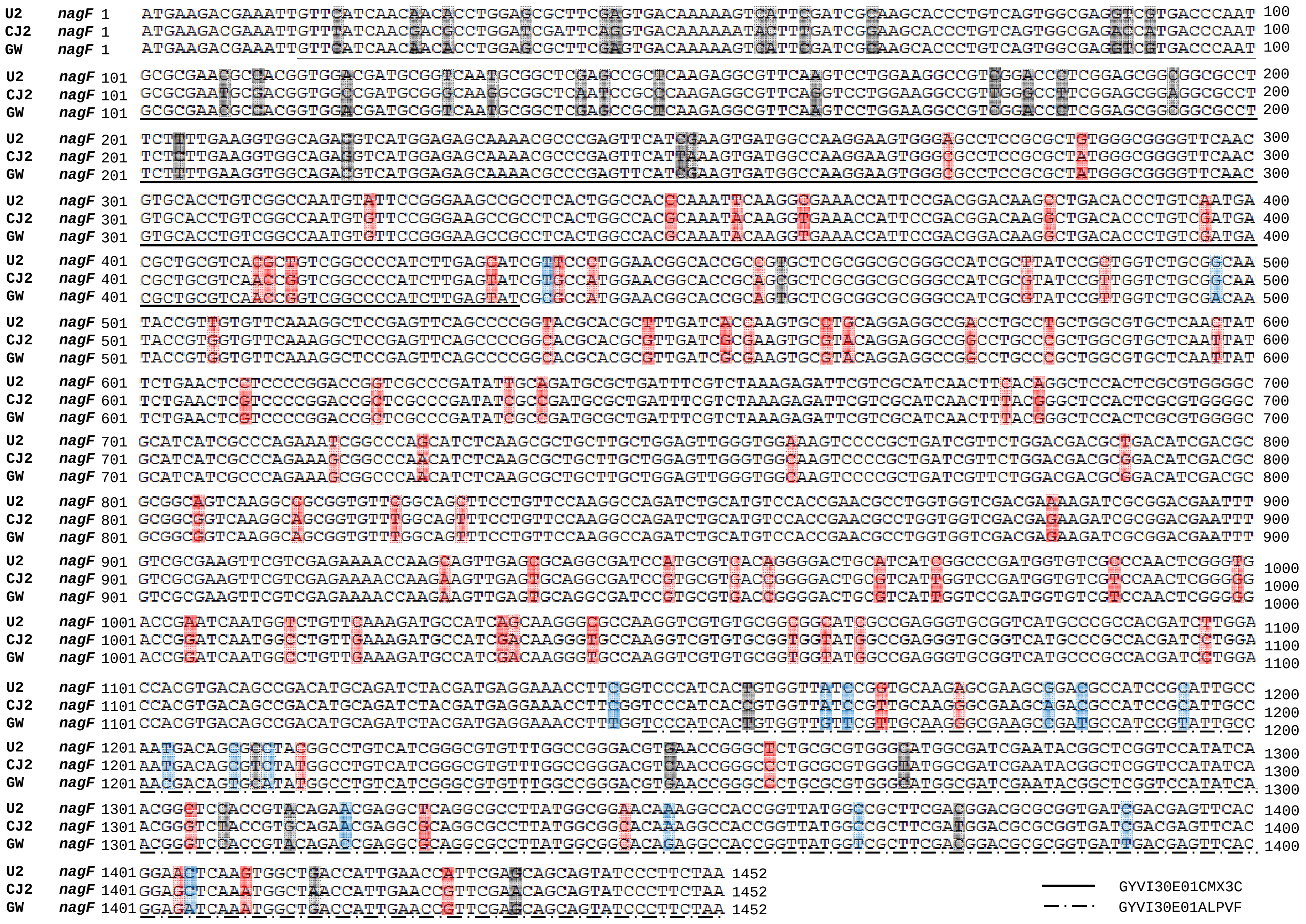
GYVI30E01CMX3C
GYVI30E01ALPVF

```
U2   nagC 1   ATGAACAAGCCAGCAACTGTCATTGAATTGGGGTACATGGCATTTCGGTCAAGGATCCCGCAGCGTGGAAATCCTTTGCCGCAAACATGCTGGGACTTC 100
CJ2  nagC 1   ATGAGCAAGCAAGCAGCAGTCATTGAACTAGGATACATGGCATTTCGGTCAAGGATCCCGCAGCGTGGAAATCCTTTGCCGCGAACATGCTGGGGCTTC 100
GW   nagC 1   ATGAACAAGCCGGCAGCAGTCATTGAATTGGGGTACATGGCATTTCGGTCAAGGATCCTGCAGCGTGGAAATCCTTTGCCGCAAACATGCTGGGACTTC 100

U2   nagC 101 AAGTCCTCGATGAGGGTGACAAGGATCGCTTCTATCTGCGAATGGACAATTGGCACCATCGGATCGTGGTTCATCACAACGGTCAAGATGACCTTGAATA 200
CJ2  nagC 101 AAGTCCTCGATGAAGGTGACAAGGACCGCTTCTATCTGCGAATGGACAATTGGCACCACAGAATCGTGGTCCATCACAATGGTCAAGATGACCTTGAATA 200
GW   nagC 101 AAGTCCTCGATGAGGGTGACAAGGATCGCTTCTATCTGCGAATGGACAATTGGCACCATCGGATCGTGGTTCATCACAACGGTCAAGATGACCTTGAATA 200

U2   nagC 201 CCTGGGCTGGCGTGTCGCCGGTCAACCGGAATTCGAGGCATTGGGTCAAAAGCTCGTGGACGCAGGCTACAAAGTCCGCGTGTGCGACAAAGCCGAAGCA 300
CJ2  nagC 201 CTTGGGTTGGCGTGTCTCCGGTCAACCGGAATTCGATGCATTGGGCCAAAAGCTCCAGGACGCAGGCTACAAAGTCCACGTGTGCGACAAAGCCGAAGCA 300
GW   nagC 201 CCTGGGCTGGCGTGTCGCCGGTCAACCGGAATTCGAGGCATTAGGTCAAAAGCTCGTGGACGCAGGCTACAAAGTCCGCGTGTGCGACAAAGCCGAAGCA 300

U2   nagC 301 CAAGAACGGATGGTGCTGGGCCTGATGAAGACAGAAGATCCGGGGGGCAACCCGACCGAGATTTTCTGGGGACCCCGGATTGACCTGAACAACCCCTTCC 400
CJ2  nagC 301 CAGGAACGCATGGTGCTGGGCCTGATGAAGACGGAAGATCCGGGTGGCAACCCGACCGAGATTTTCTGGGGACCCCGGATAGACCTGAACAACCCTTTCC 400
GW   nagC 301 CAAGAACGGATGGTGCTGGGCCTGATGAAGACAGAAGATCCGGGGGGCAACCCGACCGAGATTTTCTGGGGACCTCGGATTGACCTGAACAATCCCTTCC 400

U2   nagC 401 ATCCCGGTCGTCCCTTGCACGGGAAATTTCTAACCGGTGATCAGGGCCTGGGCCACTGCATCGTGCGTCAGAACGATGTTGAAGCGGCACGTAAGTTCTA 500
CJ2  nagC 401 ACCCCGGTCGCCCCTTGCACGGCAAGTTCTTGACTGCTGACCAAGGCCTGGGCCACTGCATAGTGCGTCAGAGCGACGTGGAAGCGGCGCGCAAGTTCTA 500
GW   nagC 401 ACCCCGGTCGTCCCTTGCACGGAAAATTTCTAACCGGCGATCAGGGCCTAGGCCACTGCATCGTGCGTCAGAATGATGTTGAAGCGGCGCGTAAGTTCTA 500

U2   nagC 501 TAGCTTGCTGGGATTTCGTGGAGATGTCGAGTACCGCCTTCCTTTGCCCAACGGCATGACGGCTGAGTTGACGTTCATGCATTGCAATGCTCGCGATCAT 600
CJ2  nagC 501 CAGCCTGCTGGGCTTCCGTGGGGATGTCGAGTACCGCCTGCCCCTGCCCAACGGCATGACCGCTGAACTGACTTTCATGCATTGCAACGCTCGCGATCAC 600
GW   nagC 501 TAGCTTGCTGGGATTTCGTGGAGATGTCGAGTACCGCCTTCCTTTGCCCAACGGCATGACGGCTGAGTTGACGTTCATGCATTGCAATGCTCGCGATCAT 600

U2   nagC 601 TCCATCGCTTTCGGTGCAATGCCTGCGGCCAAGCGCCTCAATCATCTGATGATTGAATACACTCATATCGAAGATTTGGGTTGCACACACCAGCTTTTCA 700
CJ2  nagC 601 TCCATCGCGTTCGGCGCAATGCCTGCGGCCAAGCGCATCAATCATCTGATGATTGAATACACCCATCTGGAAGATCTGGGTTATACGCACCAGCTTTTCA 700
GW   nagC 601 TCCATCGCTTTCGGTTCAATGCCTGCGGCCAAGCGCCTCAATCATCTGATGATTGAATACACTCATATCGAAGATTTGGGTTACACACACCAGCTTTTCA 700

U2   nagC 701 CGAAGGAAAAGATTGACATTGCCTTGCAATTGGGCATCCATTCCAACGATAAGGCGCTGACGTTCTACGGGGCAACACCTTCCGGCTGGCTGATAGAACC 800
CJ2  nagC 701 TGAAGAACAAGATTGACATCGCATTGCAACTGGGTATTCATGCCAACGACAAGGCGCTGACGTTCTATGGTGCAACACCTTCCGGCTGGCTGATCGAACC 800
GW   nagC 701 CGAAGGAAAAGATTGACATTGCCTTGCAATTGGGCATCCATTCCAACGATAAGGCGCTGACGTTCTACGGGGCAACACCTTCCGGCTGGCTGATCGAACC 800

U2   nagC 801 TGGGTGGCGAGGCGCCCCCGCCATTGCTGAATCGGAATATTACGTCGGCGACATTTTCGGCCACACCATCGAGGCCACCGGTTATGGATTGGACGTCAAA 900
CJ2  nagC 801 TGGGTGGCGAGGGGCTCCCTCCATGGCCGAATCGGAATATTACGTCGGCGACATCTTCGGCCATGCTATCGAGGCCACAGGATATGGTTTGGACGTCAAG 900
GW   nagC 801 TGGGTGGCGAGGGGCTCCCTCCATGGCCGAATCGGAATATTACGTCGGCGACATCTTCGGCCATGCTATCGAGGCCACAGGATATGGTTTGGACGTCAAG 900

U2   nagC 901 CTGAGCTAG 909
CJ2  nagC 901 CTGAGCTAA 909
GW   nagC 901 CTGAGCTAA 909
```

——— GYVI30E01B3SLS

—·—·— GYVI30E01CD7IX

```
U2   nagQ 1   ATGATCAAAGAAGCCATTTCCCTCGCAGGACTTGGGATGCTGATGCTCAGCACCGCATATGCCGAGGATTCCCGTTGGTCATATCGCATCGGCGCCACCA 100
CJ2  nagQ 1   ATGATCAAAAAAGCCATTTCCCTCGCCGCGGTTGGGATGCTGATGCTCAGCCATGCATATGCCGAGGATTCCCGCTGGTCCTACCGCATCGGTGCCACCA 100
GW   nagQ 1   ATGATCAAAAAAGCCATTTCCCTCGCCGCGGTTGGGATGCTGATGCTCAGCCATGCATATGCCGAGGATTCCCGCTGGTCCTACCGCATCGGTGCCACCA 100

U2   nagQ 101 ACGTAGCCTTCGATGCGAGCGCCAAAGTTTCGATTGACGGAACAAGGGTGCCGGGTGGAAGCGCTGACGCCAGTGACAACAACGCTTTGACATTTGACTT 200
CJ2  nagQ 101 ACGTGGCCTTCGATGCGAGCGCCGAAGTCTCTATGAATGGAGGGAGAGTGCCGGGAGGCAGTGCCGACGCCAGCGACGACAACGCTTTGACGTTTGATTT 200
GW   nagQ 101 ACGTGGCCTTCGATGCGAGCGCCGAAGTCTCTATGAATGGAGGGAGAGTGCCGGGAGGCAGTGCCGACGCCAGCGACAACAACGCTTTGACATTTGATTT 200

U2   nagQ 201 CGGTTACATCATTAACGATAACTGGAACGCGCGATTAATTGTTGGCATTCCACCCACCACAAAAGTGACGGGCGCAGGCACGCTGCCTCCGATTTTGCTG 300
CJ2  nagQ 201 CGGCTATGTCATCAACGACAGCTGGAACGCGCGGTTGATCGTTGGCATTCCTCCCACCACAAAAGTGACGGGCGCAGGAACGCTGCCCCCCATTTTGTTG 300
GW   nagQ 201 CGGCTATGTCATCAACGACAGCTGGAACGCGCGGTTGATCGTTGGCATTCCTCCCACCACAAAAGTGACGGGCGCAGGAACGCTGCCCCCCATTTTGTTG 300

U2   nagQ 301 GGCCGTGTCCAATATGCTCCTGCAGTTTTGTCGGCGACCTACAACCTGCCACAGATGGGATTGGTTCGCCCGTACGTGGGGGCGGGGATCAACTACACTC 400
CJ2  nagQ 301 GGCCGCATTCAATACGCCCCTGCGGTTTTGTCGGTGACCTACAACCTGCCTCAGATGGGGGGATTCGCCCTTACGTGGGGGCGGGGATCAACTACACCC 400
GW   nagQ 301 GGCCGCATTCAATACGCCCCTGCGGTTTTGTCGGTGACCTACAACCTGCCTCAGATGGGGGGATTCGCCCTTACGTGGGGGCGGGGATCAACTACACCC 400

U2   nagQ 401 GAATTCTGAAAAGCAAAGATGCCAATCTGACCTCATTCGATGCAGATCATGCGTGGGCACTTGTGCTCCACATCGGTGCAGAAGCGAACATCAACCGCGA 500
CJ2  nagQ 401 GAATTTTAAAAAGCGAAGACGCCAATCTGACCTCATTCGATGCAGATCATGCTTGGGCACCTGTGCTCCACATCGGCGCGGAAGCCGACATCAACCGCGA 500
GW   nagQ 401 GAATTTTAAAAAGCGAAGACGCCAATCTGACCTCATTCGATGCAGATCATGCTTGGGCACCTGTGCTCCACATCGGCGCGGAAGCCGACATCAACCGCGA 500

U2   nagQ 501 CTGGTTCGTCAGCTTTGATATCCGAAAACTTTATCTAAAAACAGATGCATCGGGGTTTCTTGGGCCTCAAGTTGCTACAGCCCGCGTAACGTTGAATCCG 600
CJ2  nagQ 501 CTGGTTTGTCAGCTTTGATCTCCGCAAACTTTATTTGAAAACAGATGCATCTGGGTTTCTTGGCCCGCAAGTCGCCACGGCCCGCGTAACATTGAATCCG 600
GW   nagQ 501 CTGGTTTGTCAGCTTTGATCTCCGCAAACTTTATTTGAAAACAGATGCATCTGGGTTTCTTGGCCCGCAAGTCGCCACGGCCCGCGTAACATTGAATCCG 600

U2   nagQ 601 CTGCTGACGTCGATTGCGATTGGCCGGAGATTCTGA 636
CJ2  nagQ 601 TTGTTGACCTCGATTGCGATTGGTCGGAGATTTTGA 636
GW   nagQ 601 TTGTTGACCTCGATTGCGATTGGTCGGAGATTTTGA 636
```

```
U2   nagE 1    ATGACAAGAAAGACGAGCAAAGCGGTGCGCCTGACCGCCGCGGATATTCAAGGCGCATGGGTCATCATGCCGACCCCGTCCACGCCGGATGCCTCGGACT 100
CJ2  nagE 1    ATGACAAGAAAGACCAGCAAAGCGACGCGCCTGACTGCAGAGGATATTCAAGGCGCATGGGTCATTATGCCGACCCCCTCCACTCCGGATGCTTCGGACT 100
GW   nagE 1    ATGACAAGAAAGACCAGCAAAGCGACGCGCCTGACTGCAGAGGATATTCAAGGCGCATGGGTCATTATGCCGACCCCCTCCACTCCGGATGCTTCGGACT 100

U2   nagE 101  GGCGCAGCACGCACACGGTCGATCTCGACGAGACGGCCCGGATTGTCGAGGAGTTGATTGCGGCCGGCGTCAATGGCATTCTGAGCCACGGCACCTTTGG 200
CJ2  nagE 101  GGCGCAGCACGCACACGGTTGATCTCGACGAGACGGCCCGGATCGTCGAGGAGTTGATAGCGGCCGGGGTCAATGGCATTCTAAGCCACGGCACTTTTGG 200
GW   nagE 101  GGCGCAGCACGCACACGGTTGATCTCGACGAGACGGCCCGGATCGTCGAGGAGTTGATAGCGGCCGGGGTCAATGGCATTCTAAGCCACGGCACTTTTGG 200

U2   nagE 201  CGAATGCGCGACGCTGACGTGGGAGGAGAAGCGGGATTTTGTTTCAACGGTCGTGGAAACCGCGCGCGGTCGAGTGCCCTACTTCTGCGGCACAACGGCC 300
CJ2  nagE 201  TGAATGCGCGACGCTGACGTGGGAAGAGAAGCGGGATTTTGTTTCAACGGTCGTCGAAACCGCCCGTGGTCGAGTGCCCTACTTCTGCGGCACAACAGCC 300
GW   nagE 201  TGAATGCGCGACGCTGACGTGGGAAGAGAAGCGGGATTTTGTTTCAACGGTCGTCGAAACCGCCCGTGGTCGAGTGCCCTACTTCTGCGGCACAACAGCC 300

U2   nagE 301  TTGAATACCCGTGAAGTCATACGCCAGACCCGCGAATTGATCGACATTGGCGCCCAAGGAACAATGCTCGGCGTGCCGATGTGGGTGAAGATGGATCTGC 400
CJ2  nagE 301  TTGAATACCCGTGAAGTTATACGCCAGACCCGCGAAGTGATGGACATTGGTGCCCAAGGAACCATGCTCGGCGTGCCGATGTGGGTGAAGATGGATCTGC 400
GW   nagE 301  TTGAATACCCGTGAAGTTATACGCCAGACCCGCGAAGTGATGGACATTGGTGCCCAAGGAACCATGCTCGGCGTGCCGATGTGGGTGAAGATGGATCTGC 400

U2   nagE 401  CTACTGCCGTGCAATTTTATCGCGATGTGGCGGAAGCAGTGCCAGATGCAGCCATCGCTGTCTACGCCAACCCGGAGGCTTTCAAATTTGATTTTCCTCG 500
CJ2  nagE 401  CCACCGCCGTGCAGTTTTATCGCGATGTGGCGGAAGCGGTGCCAGATGCTGCCATCGCTGTTTATGCCAACCCGGAGGCTTTCAAATTTGATTTTCCTCG 500
GW   nagE 401  CCACCGCCGTGCAGTTTTATCGCGATGTGGCGGAAGCGGTGCCAGATGCTGCCATCGCTGTTTATGCCAACCCGGAGGCTTTCAAATTTGATTTTCCTCG 500

U2   nagE 501  CCCGTTTTGGGCCGAAATGTCCAAAATCCCGCAGGTTGTCACAGCCAAGTACTTGGGCATCGGGATGCTGGACTTGGATCTGAAATTGGCCCCAAATATT 600
CJ2  nagE 501  CCCGTTTTGGGCCGAAATGTCCAAAATCCCGCAAGTTGTTACAGCCAAGTACTTGGGCATCGGAATGCTGGACTTGGATCTGAAATTGGCTCCAAATATT 600
GW   nagE 501  CCCGTTTTGGGCCGAAATGTCCAAAATCCCGCAAGTTGTTACAGCCAAGTACTTGGGCATCGGAATGCTGGACTTGGATCTGAAATTGGCTCCAAATATT 600

U2   nagE 601  CGCTTCCTTCCGCATGAGGATGACTACTACGCTGCGGCCCGGATCAATCCCGAGCGCATGACTGCTTTCTGGTCTAGCGGTTCCATGTGCGGCCCAGCGA 700
CJ2  nagE 601  CGCTTCCTTCCGCATGAGGATGACTATTACGCTGCGGCCCGAATCAATCCTGAGCGCATGACCGCCTTCTGGTCCAGCGGCTCCATGTGCGGTCCAGCGA 700
GW   nagE 601  CGCTTCCTTCCGCATGAGGATGACTATTACGCTGCGGCCCGAATCAATCCTGAGCGCATGACTGCTTTCTGGTCTAGCGGTTCCATGTGCGGCCCAGCGA 700

U2   nagE 701  CCGCCCTTGTGCTGCGCGATGAGGTGGTAAAGGCCAAAAATACAGGTGATTGGGCCAAGGCCAAGGCTATTTCAGATGACATGCGCGCAGCCGATGCCAC 800
CJ2  nagE 701  CTGCCCTCATGCTTCGTGATGCGGTGACACAGGCCAAGACGACCGGTGACTGGACCAAGGCCAAGGCCATTTCAGATGACATGCGCGCTGCCGACGCCAC 800
GW   nagE 701  CCGCCCTTGTGCTGCGCGATGAGGTGGTAAAGGCCAAAAATACAGGTGATTGGGCCAAGGCCAAGGCTATTTCAGATGACATGCGCGCAGCCGACGCCAC 800

U2   nagE 801  ACTGTTTCCACGCGGCGATTTCTCGGAATTCTCAAAATACAACATTGGCCTCGAAAAAGCACGAATGGACGAGGCCGGCTGGCTCAAGGCGGGGCCGTGC 900
CJ2  nagE 801  ACTGTTTCCACGCGGCGATTTCTCGGAATTCTCGAAATACAACATTGGTCTCGAAAAAGCACGAATGGACGAGGCGGGTTGGCTCAAAGCGGGGCCGTGC 900
GW   nagE 801  ACTGTTTCCACGCGGCGATTTCTCGGAATTCTCAAAATACAACATTGGCCTCGAAAAAGCACGAATGGACGAGGCCGGCTGGCTCAAGGCGGGGCCGTGC 900

U2   nagE 901  CGGCCACCCTATACGCTGGTTCCCGACGAATACCTTGCAGGTGCCCGAAAATCAGGCAAGGCCTGGGCCGCACTGCATACCAAGTATGCCAAGGAATTGA 1000
CJ2  nagE 901  CGGCCACCCTATACGCTGGTACCAGATGAATACCTTGCAGGTGCTCGAAAATCAGGCAACGCCTGGGCCGCACTGCATACCAAGTACGCCGAGGAATTGA 1000
GW   nagE 901  CGGCCACCCTATACGCTGGTTCCCGACGAATACCTTGCAGGTGCCCGAAAATCAGGCAAGGCCTGGGCCGCACTGCATACCAAGTATGCCAAGGAATTGA 1000

U2   nagE 1001 GGAAAACCAAAACGGCAACCAACTCGAAAAAGAAGTAA 1038
CJ2  nagE 1001 AGAAAACCAAAACGGCAACCGGCTCGAAAAAGAAGTGA 1038
GW   nagE 1001 GGAAAACCAAAACGGCAACCAACTCGAAAAAGAAGTAA 1038
```

GYVI30E01EB2RY

GYVI30E01EQ8UF

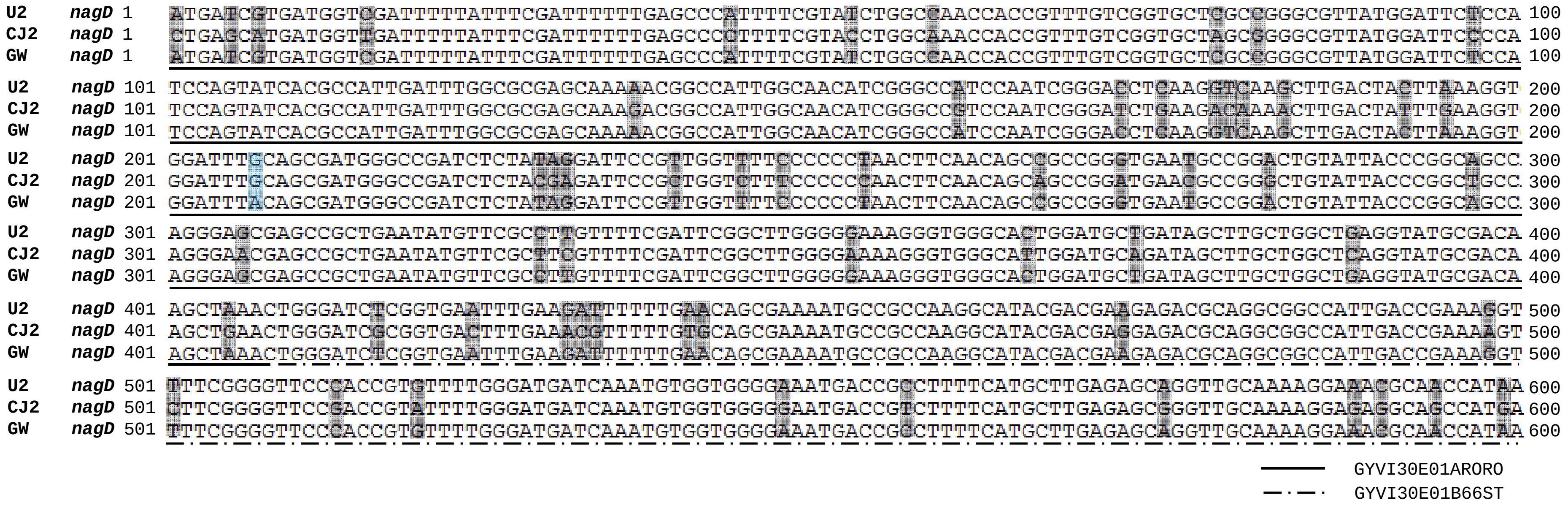

Figure S6. Multi-sequence alignment of the *nag* operons, including *nag2*, *nag*-U2 and *nag*-CJ2.
Bases which were not the same between *nag2*, *nag*-U2 and *nag*-CJ2 are colored.
Grey hightlight represents homologous bases between *nag2* and *nag*-U2, but different from *nag*-CJ2;
Red highlight represents homologous bases between *nag2* and *nag*-CJ2, but different from *nag*-U2;
Blue highlight represents bases of *nag2* with no homologies with either *nag*-CJ2 or *nag*-U2.
The DNA sequence of *nagFCQED* is from the plasmid pWH_NagFCQED. Bases from single 454 sequencing read are underlined and marked alongside each gene. The numbers on the bottom right are the codes of individual 454 sequence reads.
