# Supplementary material for: A Culture-Independent Approach to Unravel Uncultured Bacteria and Functional Genes in a Complex Microbial Community
Source: PLoS One. 2012 Oct 17;7(10):e47530. doi: 10.1371/journal.pone.0047530 (PMC3474725; doi:10.1371/journal.pone.0047530)
Supplement: Figure S7 — A single pyrosequencing read in the 13C-DNA fraction links nagB and nagF in nag2 operon. (PDF) [file pone.0047530.s007.pdf]

>GYVI30E01D06VU length=506 xy=1536_2696 Rev com

TTTCTTCCTATGCGGTCGGTGGCGGCGGTTCTTGCTACATGGCCAGCAA
ACATGCGGTGCTGGGCATGGTCAAGGCTTTGGCCTACGAATTGGCTCCG
CACATCCGGGTCAATGGCGTTGCGCCAGGTGGTGCGGTCACTTCTTTGG
CTGGCCCGGCAAGCGCTGGCTTCGACAAAACCAAAATGAAAGACATGCC
CGGCATCGATGACATGATCAAAGGCCTGACTCCCCTGGGGTTCGCGGCA
AGGCCCGAGGACGTGGTGGCACCGTATCTTTTGCTGGCCTCCCGGGAAC
AAGGGAAGTTCATCACTGGCACCGTAATCGGCATTGATGGCGGCATGGC
GCTCGGTCGAAAGTGA(nagB)ATTTTCAATCAAATCAGATTTTCAACC
CCATTCCCAGGAGACAACCCATGAAGACGAAATTGTTCATCAACAACAC
CTGGAGCGCTTCGAGTGACAAAAAGTCATTCGATCGCAAGCACCCTGTC
AGTGGCGAGGTCGTGACCCAAT(nagF)

Figure S7. A single pyrosequencing read in the ^{13}C-DNA fraction links *nag*B and *nag*F in nag2 operon.
